# Directed information flow during laparoscopic surgical skill acquisition dissociated skill level and medical simulation technology

**DOI:** 10.1038/s41539-022-00138-7

**Published:** 2022-08-25

**Authors:** Anil Kamat, Basiel Makled, Jack Norfleet, Steven D. Schwaitzberg, Xavier Intes, Suvranu De, Anirban Dutta

**Affiliations:** 1 Center for Modeling, Simulation and Imaging in Medicine, Rensselaer Polytechnic Institute, Troy, NY USA; 2 US Army Futures Command, Combat Capabilities Development Command Soldier Center STTC, Orlando, FL USA; 3 University at Buffalo School of Medicine and Biomedical Sciences, Buffalo, NY USA; 4 Department of Biomedical Engineering, Rensselaer Polytechnic Institute, Troy, NY USA; 5 Neuroengineering and Informatics for Rehabilitation Laboratory, Department of Biomedical Engineering, University at Buffalo, Buffalo, NY USA

**Keywords:** Education, Sensorimotor processing

## Abstract

Virtual reality (VR) simulator has emerged as a laparoscopic surgical skill training tool that needs validation using brain–behavior analysis. Therefore, brain network and skilled behavior relationship were evaluated using functional near-infrared spectroscopy (fNIRS) from seven experienced right-handed surgeons and six right-handed medical students during the performance of Fundamentals of Laparoscopic Surgery (FLS) pattern of cutting tasks in a physical and a VR simulator. Multiple regression and path analysis (MRPA) found that the FLS performance score was statistically significantly related to the interregional directed functional connectivity from the right prefrontal cortex to the supplementary motor area with *F* (2, 114) = 9, *p* < 0.001, and *R*^2^ = 0.136. Additionally, a two-way multivariate analysis of variance (MANOVA) found a statistically significant effect of the simulator technology on the interregional directed functional connectivity from the right prefrontal cortex to the left primary motor cortex (*F* (1, 15) = 6.002, *p* = 0.027; partial *η*^2^ = 0.286) that can be related to differential right-lateralized executive control of attention. Then, MRPA found that the coefficient of variation (CoV) of the FLS performance score was statistically significantly associated with the CoV of the interregionally directed functional connectivity from the right primary motor cortex to the left primary motor cortex and the left primary motor cortex to the left prefrontal cortex with *F* (2, 22) = 3.912, *p* = 0.035, and *R*^2^ = 0.262. This highlighted the importance of the efference copy information from the motor cortices to the prefrontal cortex for postulated left-lateralized perceptual decision-making to reduce behavioral variability.

## Introduction

Virtual reality (VR) technology is increasingly being used for motor skill training in medicine^1^; however, the investigation of the brain–behavior relationship in VR compared to physical simulators is lacking. Specifically, there is a need to study directional information flow in the brain during interactions with the environment for motor skill acquisition. During motor skill acquisition, the goal of the interactions with the environment is postulated to build models (called internal models^2,3^) that can take the desired trajectory and output the corresponding motor command (i.e., inverse models) or take an efferent copy of motor command and predict the future state of the body (i.e., forward models)^4^ for perceptual decision-making to reduce behavioral variability. Internal models can then be used for sensory processing, sensorimotor integration, and motor control leading to expert performance. For example, specific actions are possible whereas others are not in an operative field in laparoscopic surgery, and the possibilities of goal-directed action in the environment^5^, called “affordances,” must be learned by novices for planning sensory-guided actions toward a goal^6,7^. The purpose of the internal models is to inform action so that the central nervous system can internally simulate possibilities of goal-directed motor behavior in planning, control, and learning^8^. Here, motor learning involves making sensory predictions from efferent copies of motor commands, performing the action, and then validating the predictions with corresponding sensory information^9^ from the environment called the “reafferent,” that is, the sensory information generated by the action and the interactions with the environment. Such “reafferent” sensory input is crucial in medical simulators for action monitoring^8^ and action-specific perception that action is the consequence of one’s intention, which is commonly referred to as agency. Christensen et al.^10^ investigated the coupling between action and perception based on the effect of action execution on action perception. These researchers found that integrating motor and multisensory information for action-specific perception depended on the cerebellum, which is thought to encode internal models^11^. Specifically, an intact forward model can predict a future state from the efferent copy of the motor command, thereby coupling action execution with action perception. Moreover, patients with cerebellar damage showed no beneficial influence of action execution on action perception compared to healthy controls, thereby showing a lack of action-perception coupling. Sensorimotor integration with the inverse model to determine the motor commands for the desired goal-directed trajectory, then action execution and action perception using the forward model for fine control of voluntary movements^11^ will require the interaction of the cerebellum with the cerebrum, i.e., the sensorimotor cortico-cerebellar loops^12^.

Motor exploration, a trial-and-error process due to a lack of adequate internal models in a novel environment for perceptual decision-making, plays a critical role in motor learning^13^. During goal-directed movement, reaching the goal is the “reward,” and actions that lead to the goal are reinforced. For example, motor behavior for laparoscopic surgery training can be characterized as a coordinated spatiotemporal three-dimensional (3D) movement using two-dimensional (2D) camera feedback with the interaction between the body and the environment within a restricted surgical volume representing a novel procedure for novices. Here, novices need to learn a complex bimanual motor task requiring high-precision hand-eye coordination, depth perception in the 2D view, and goal-directed tool control for optimal performance of laparoscopic surgery^14^. Although there can be more than one trajectory of goal-directed movement^15^, there are only a few “efficient” trajectories, and not everyone can achieve proficiency^16^. For example, novices may switch early from motor exploration to motor exploitation^17^, where they reinforce “inefficient” trajectories despite errors for goal-directed movement. Indeed, motor skill automaticity, i.e., a decrease in the need for effortful control over performance^18^, can be achieved despite the residual error. Therefore, an adequate switch from motor exploration to motor exploitation^17^ during the motor learning process is important^13^, where explore-exploit decisions are increasingly being shown to be dependent on behavioral variability^19^. Computational modeling of the interplay between the cerebellum and basal ganglia in motor adaptation^20^ predicts that the learner is less likely to adapt to perturbations when the expected movement variability is “low,” and an underestimated variability can lead to an overly strong reduction in the learning rate for small perturbations. Here, switching to nonerror-based basal ganglia learning mechanisms can also be due to the implicit cost of error correction for the brain^21^; however, acquiring expert performance will require deliberate practice^22^ with overestimated variability. Deliberate practice^22^ is postulated to drive error-based cerebellar learning mechanisms^20^ despite the cost of perfecting the internal model for perceptual decision-making to reduce behavioral variability.

In this brain–behavior study, we aimed to capture the neural correlates of behavioral variability in terms of neural variability^23^. Development of the sensorimotor mapping during skill acquisition is usually under variability “for all sets or series of observations that are nonconstant and … nonstationary”^24^, including variability in brain activation^25^. The inferences about the state of the tool and the environment under noisy feedback will be made using a perceptual model that novices need to develop based on the prediction error during the trial-and-error process of motor exploration. Therefore, understanding the variability in the brain and behavior in the context of the perception-action cycle^26^ can provide insights into individual exploration–exploitation trade-offs in human motor learning^27^. Moreover, it is important for motor skill training in medicine^1^ to monitor brain–behavior relationships to identify novices who can learn more efficiently during the basal ganglia-driven motor exploration stage^27^. Indeed, mobile brain–behavior investigation during motor skill training in medicine^1^ is feasible due to recent developments in portable brain-imaging technologies^28^. For example, Nemani et al.^29^ assessed bimanual motor skills using functional near-infrared spectroscopy (fNIRS) during laparoscopic surgery training. In this study, we investigated the brain–behavior relationship in the context of the perception-action cycle^26^ based on the directional information flow in the novice brain compared to the expert brain.

Laparoscopic surgery training following the Fundamentals of Laparoscopic Surgery (FLS) is a common education and training module designed for medical residents, fellows, and physicians to provide them with a set of basic surgical skills necessary to conduct laparoscopic surgery successfully. The FLS training is a joint education program between the Society of American Gastrointestinal Endoscopic Surgeons and the American College of Surgeons to establish box trainers (physical simulators) in standard surgical training curricula^30^. FLS certification in general surgery in the USA involves five psychomotor tasks with increasing task complexity: (i) pegboard transfers, (ii) pattern cutting, (iii) placement of a ligating loop, (iv) suturing with extracorporeal knot tying, and (v) suturing with intracorporal knot tying. This certification was introduced to systemize the training and evaluation of cognitive and psychomotor skills required to perform minimally invasive surgery. FLS is being used to measure and document these skills for medical practitioners, where the understanding of the brain–behavior relationship is crucial for informed training and assessment^31^, especially in the context of physical versus VR simulators during laparoscopic surgery training^32^. For example, surgeons rely on 2D visualization of the 3D surgical field at a reduced depth and tactile perception^33^, where 3D vision has been shown to speed up laparoscopic training^34^ potentially by reducing the perceptual load^35^. Therefore, understanding the perception-action coupling from the brain–behavior analysis can be used to improve VR simulators to help novices learn more efficiently with exploration–exploitation trade-offs in human motor learning^27^. For example, behavioral variability can be artificially modulated in VR simulators^20^ to modulate the exploration–exploitation trade-off in human motor learning^27^ that can be individualized based on portable brain imaging of error processing mechanisms^36^. However, VR-driven sensorimotor stimulation may also have harmful aftereffects^37^, impeding transfer to real-life conditions that can be probed with the brain–behavior relationship in the context of sensation weighting in the perception-action cycle^38^, e.g., a greater reliance on visual feedback due to the lack of realistic kinesthetic and tactile feedback in VR simulators. Therefore, understanding the perception-action cycle from the brain–behavior analysis can aid in improving the design of VR simulators in medicine, e.g., providing adequate sensory information with kinesthetic and tactile feedback^1,39^ in addition to realistic visual and auditory feedback guided by portable brain imaging.

**Fig. 1:**
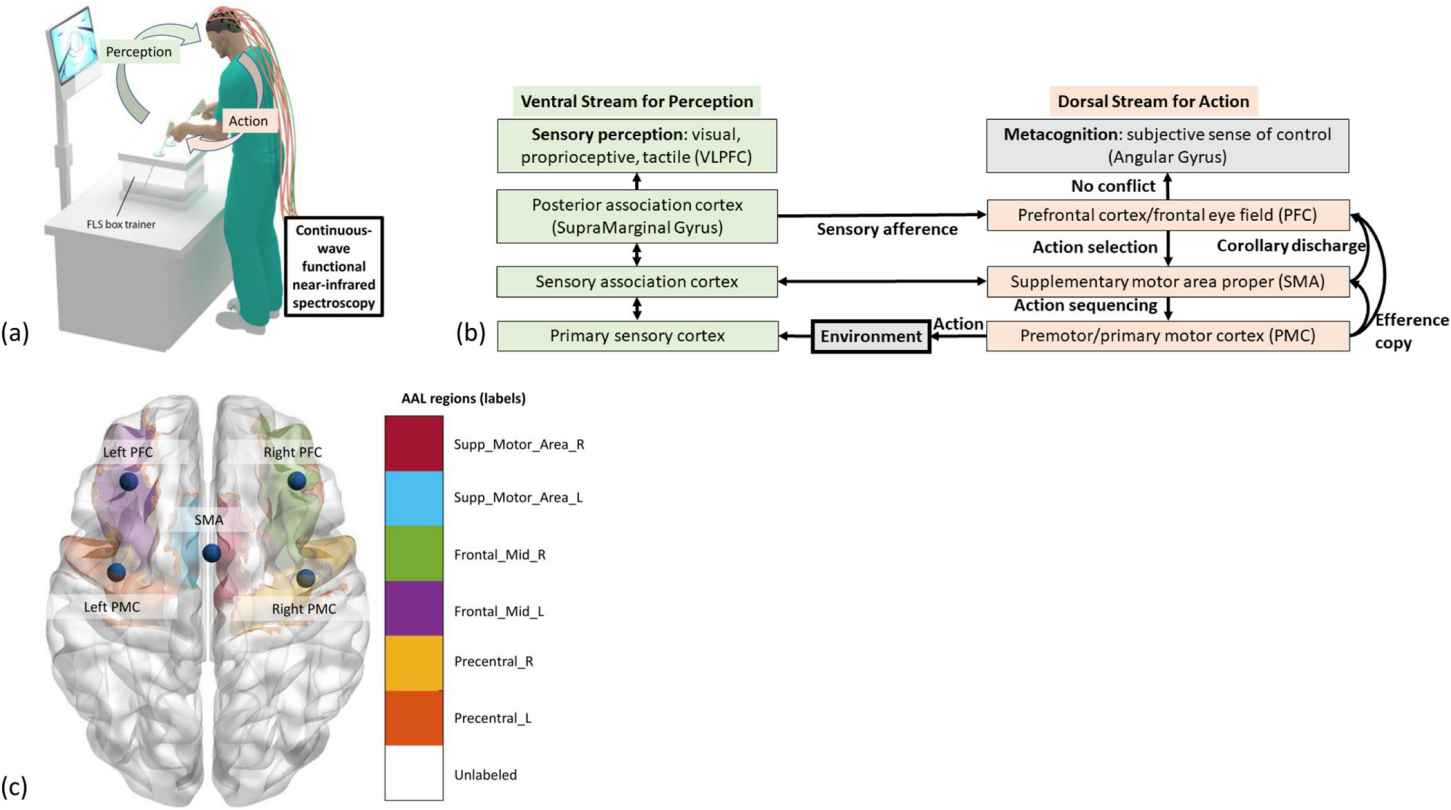
Experimental setup. **a** Subject performing the bimanual FLS task while a continuous-wave spectrometer is used to simultaneously measure functional brain activation via functional near-infrared spectroscopy to capture the perception-action link to the surgical training. **b** Perception action model for surgical training in the physical and VR simulator environments. Our portable neuroimaging allowed investigation of the dorsal stream of action in the following brain regions, including the right PFC (RPFC), left PFC (LPFC), SMA, right PMC (RPMC), and left PMC (LPMC), based on the sensitivity profile of our optode montage (Supplementary Fig. 1). This study did not investigate the ventral stream for perception, including the ventrolateral PFC (VLPFC) region, based on the sensitivity profile of our optode montage (Supplementary Fig. 1). **c** Automated anatomical labeling (AAL) of the brain regions (see Supplementary Table 1), Supp_Motor_Area_R (SMA), Supp_Motor_Area_L (SMA), Frontal_Mod_R (RPFC), Frontal_Mod_L (LPFC), Precentral_R (RPMC), Precentral_L (LPMC) based on the optode sensitivity profile (Supplementary Fig. 1).

The FLS task performance (see Fig. 1a) is graded based on the speed and accuracy related to psychomotor skills^40^, where a speed-accuracy trade-off during skill training can lead to automaticity with a greater focus on speed despite residual error^18,41^, i.e., an increased speed of action selection with “inefficient” movement trajectories. Here, the early motor learning phase recruits the cerebral structures involving the striatum (nuclear complex of the basal ganglia) and the cerebellum, and the interaction between these two structures is thought to be critical for learning new movement trajectories^42^. Notably, the cerebellum seems to provide a substrate for error-based learning through updating a forward model, whereas the striatum underpins habit formation (related to automaticity)^42^. Therefore, we postulate that successful motor skill acquisition requires adequate updating of a forward model in the cerebellum^43^ during basal ganglia-driven motor exploration that will be reflected in the directional information flow in the brain cortico-basal ganglia-cerebellar network^44^. Then, the hierarchy of cognitive control during skill learning shows a rostrocaudal axis in the frontal lobe^45^, where a shift from posterior to anterior is postulated to mediate the progressively abstract, higher-order control expected from experts. Numerous functional magnetic resonance imaging (fMRI) and fNIRS studies have been published on skill learning^33,46–53^, including training under stress^54^; however, these studies have not systematically investigated the directional cortical information flow^55,56^, its variability during FLS skill acquisition in physical versus VR simulators, and its interaction with the skill level based on statistical path analysis^57,58^.

In this study, we used fNIRS-based brain imaging, given that fMRI is challenging for mobile brain–behavior studies of FLS skill acquisition. Published fNIRS studies on skill learning showed the involvement of the inferior parietal cortex, prefrontal cortex (PFC), occipital cortex, and sensorimotor areas, including the premotor and primary motor cortex (PMC), whereas fMRI studies showed additional activation of deeper brain structures, including the basal ganglia and cerebellum^33,46–53^, and a large-scale brain network^59,60^. Here, fNIRS-based brain imaging has limited spatial and depth sensitivity^61^, which needs to be considered for brain–behavior analysis. Then, the directional information flow^55,56^ can be elucidated based on dynamic functional brain connectivity^62^ that measures time-varying changes in cortical activation and their dynamic reconfiguration^63^. Specifically, directed functional brain connectivity based on time-varying Granger causality analysis^64^ can be used to elucidate directional information flow across brain regions in the context of motor exploration (novices) versus exploitation (experts) underlying perception-action coupling^27^.

Our prior work^65^ established the face and construct validity of the VR simulator used in this study. Additionally, our previous mobile brain–behavior study found wavelet coherence-based interhemispheric primary motor cortex connectivity and its coefficient of variation (CoV) to be different between physical and VR simulators in novices^66^; however, the analysis of directed information flow^67^ to elucidate the hierarchy of the related to the perception-action cycle^68,69^ (Fig. 1b) was not performed. Therefore, we investigated the directed information flow^67^ among the following brain regions: the right PFC (RPFC), left PFC (LPFC), supplementary motor area (SMA), right PMC (RPMC), and left PMC (LPMC) (see Fig. 1b). These regions were identified based on the sensitivity profile of our optode montage (Supplementary Fig. 1). Figure 1b shows the postulated perception-action link where our optode montage captured the dorsal stream for action starting from action selection in dorsolateral PFC (drives random exploration in novices^23^) to action sequencing in the SMA to action performance in the PMC. Then, the efference copy information from the PMC is transmitted to the SMA and PFC, whereas the corollary discharge from the SMA is transmitted to the PFC. Here, we distinguished between efference copy versus collateral discharge based on whether the motor action was transmitted versus the motor plan for action-perception^70^ in the PFC^71^. Then, any conflict with the sensory reafference is monitored by the angular gyrus for a subjective sense of agency^72^ in the simulation environment. The ventral stream for the perception of the sensory feedback from the environment at the primary sensory cortex flows to the sensory association cortex and then to the posterior association cortex (e.g., supramarginal gyrus), leading to conscious perception in the ventrolateral PFC (VLPFC). Here, the PFC interacts through reciprocal and reentrant connections with different areas of the posterior association cortex^73^, including the supramarginal gyrus, to integrate the information from multiple sensory inputs and motor actions^74^ for action-perception^70^. Figure 1c shows the automated anatomical labeling (AAL)^75^ of the brain regions with Montreal Neurological Institute (MNI) coordinates (see Supplementary Table 1) based on the sensitivity profile (Supplementary Fig. 1).

## Results

### Interregional directed functional brain connectivity

Repeated-measure two-way multivariate analysis of variance (two-way MANOVA) found a statistically significant effect of skill level (expert, novice) on interregional directed functional connectivity from the RPFC to SMA (*F* (1, 15) = 6.045, *p* = 0.027; partial *η*^2^ = 0.287), LPMC to SMA (*F* (1, 15) = 7.892, *p* = 0.013; partial *η*^2^ = 0.345) and SMA to LPFC (*F* (1, 15) = 6.591, *p* = 0.021; partial *η*^2^ = 0.305). Additionally, two-way MANOVA found a statistically significant effect of the simulator technology (physical simulator, VR simulator) on the interregional directed functional connectivity from the RPFC to LPMC (*F* (1, 15) = 6.002, *p* = 0.027; partial *η*^2^ = 0.286). Then, two-way MANOVA found a statistically significant effect of the interaction between the skill level and the simulator technology on the interregional directed functional connectivity from the LPMC to RPFC (*F* (1, 15) = 8.523, *p* = 0.011; partial *η*^2^ = 0.362) and SMA to LPFC (*F* (1, 15) = 6.824, *p* = 0.020; partial *η*^2^ = 0.313). The details of the between-subject effects are presented in Supplementary Table 2.

**Fig. 2:**
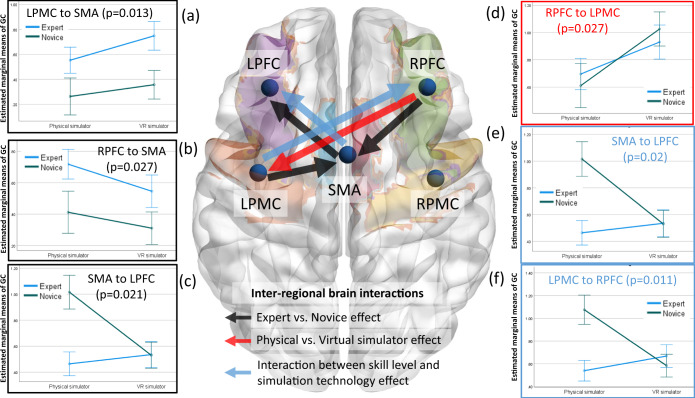
Plots of the estimated marginal means of the significant interregional directed functional brain connectivity. Error bars show the standard error. **a** Significant (*p* = 0.013) effect of skill level (expert, novice) on LPMC- to SMA-directed functional connectivity. **b** Significant (*p* = 0.027) effect of skill level (expert, novice) on RPFC- to SMA-directed functional connectivity. **c** Significant (*p* = 0.021) effect of skill level (expert, novice) on SMA to LPFC directed functional connectivity. **d** Significant (*p* = 0.027) effect of the simulator technology (physical simulator, VR simulator) on RPFC-to-LPMC-directed functional connectivity. **e** Significant (*p* = 0.020) effect of the interaction between skill level and the simulator technology on SMA to LPFC directed functional connectivity. **f** Significant (*p* = 0.011) effect of the interaction between skill level and the simulator technology on LPMC-to-RPFC-directed functional connectivity. Significant (after controlling for a false discovery rate of 0.05 with Benjamini–Hochberg adjustment) interregional directed functional brain connectivity is shown with colored arrows for the factors, the skill level (expert, novice), simulator technology (physical simulator, VR simulator), and their interactions.

Figure 2 shows the mean response for each factor (shown with colored arrows) adjusted for other variables in the model, i.e., the plot of estimated marginal means of the significant interregional directed functional brain connectivity after controlling the false discovery rate of 0.05 with Benjamini–Hochberg adjustment. Figure 2a, b, c show the plot of estimated marginal means of the interregional directed functional brain connectivity affected by the skill level (expert, novice), where efference copy information flows from LPMC to SMA and the attentional control from RPFC to SMA, both postulated for proficient sequencing of motor subtasks, higher in experts than novices across both simulators. Figure 2d shows the plot of estimated marginal means of the interregional directed functional brain connectivity affected by the simulator technology (physical simulator, VR simulator). Here, the higher interregional directed functional brain connectivity from the RPFC to LPMC in the VR simulator than in the physical simulator may be related to increased attentional processes^76^ (or attentional control) for motor control by the LPMC of the right-handed subjects since the RPFC optodes were over the right middle frontal gyrus (see Supplementary Table 1). However, the interregional directed functional brain connectivity from the RPFC to SMA trended toward being lower (Fig. 2b) in the VR simulator compared with the physical simulator, which may underpin lesser visuomotor attentional control of SMA in the VR. Additionally, the interregional directed functional connectivity from LPMC to SMA trended toward being higher (Fig. 2a) in the VR simulator than in the physical simulator, which may underpin a more substantial efference copy to SMA in the VR. Here, the VR simulator may have required lesser executive control of attention for proficient sequencing of motor subtasks than the physical simulator, which needs further investigation. The interregional directed functional connectivity from the SMA to LPFC, which is considered the corollary discharge for action-perception^70^ in PFC^71^, trended toward lower in the VR simulator than in the physical simulator for the novice and attained a similar level as that of the expert (see Fig. 2c). Notably, the VR simulator was novel for both the expert and the novice, given that experts were experienced with the physical simulator and human surgery, so a similar interregional directed functional connectivity from the SMA to LPFC in the VR simulator was expected and found, as shown in Fig. 2c. Moreover, the interregional directed functional connectivity from the LPMC to RPFC, which is considered the efference copy for executive control of attention in the right-lateralized PFC^71^, decreased in the VR simulator compared to the physical simulator for novices and attained a similar level as that of the expert (see Fig. 2e). These findings suggest a more substantial efference copy to SMA (for proficient sequencing of motor subtasks) and lesser to RPFC (for executive control of attention) in the VR simulator than the physical simulator where the efference copy contributions were comparable between experts and novices in the VR simulator.

### FLS task performance score and coefficient of variation (CoV) of the FLS task performance score

**Fig. 3:**
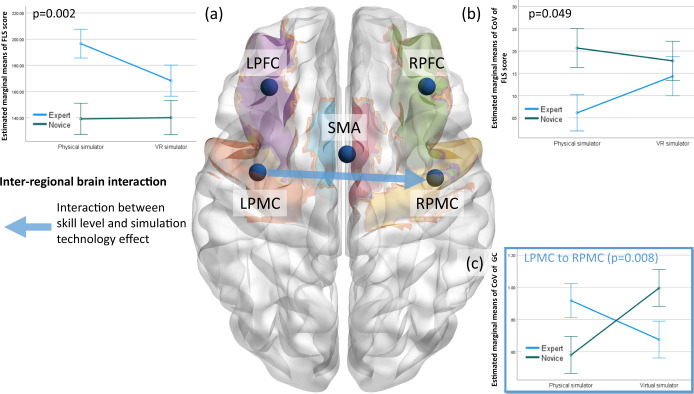
Brain-behavior analysis based on the coefficient of variation. Estimated marginal means of **a** significant (*p* = 0.002) effects of skill level on the FLS performance score and **b** significant (*p* = 0.049) effects of skill level on the coefficient of variation (CoV) of the FLS performance score. **c** Significant (*p* = 0.008) effect of the interaction between skill level and the simulator technology on the coefficient of variation (CoV) of LPMC- to RPMC-directed functional connectivity. Error bars show the standard error. Significant (after controlling for a false discovery rate of 0.05 with Benjamini–Hochberg adjustment) interregional directed functional brain connectivity is shown with a blue arrow for the interaction between skill level and the simulator technology on the CoV.

Repeated-measures two-way analysis of variance (ANOVA) found a statistically significant effect of skill level on the FLS score (*F* (1, 20) = 12.786, *p* = 0.002; partial *η*^2^ = 0.390). Supplementary Table 4 presents the tests of between-subject effects. Two-way ANOVA also found a statistically significant effect of skill level on the CoV of the FLS score (*F* (1, 21) = 4.370, *p* = 0.049; partial *η*^2^ = 0.172). Supplementary Table 5 presents the tests of between-subject effects. Figure 3a shows that the FLS score of the experts decreased in the VR simulator compared with the physical simulator given that experts were experienced only with the physical simulator and human surgery. Figure 3b shows that the CoV of the FLS score of experts increased in the VR simulator compared to the physical simulator.

### Coefficient of variation (CoV) of interregional directed functional brain connectivity

Two-way MANOVA found a statistically significant effect of the interaction between the skill level and the simulator technology on the CoV of the interregional directed functional connectivity from LPMC to RPMC (*F* (1, 21) = 8.561, *p* = 0.008; partial *η*^2^ = 0.290). Supplementary Table 3 presents the tests of between-subject effects. Figure 3c shows that the CoV of the interhemispheric directed functional connectivity from LPMC to RPMC increased in the VR simulator compared with the physical simulator for novices but decreased for the experts. Here, the CoV of the FLS score of experts increased in the VR simulator compared with the physical simulator (Fig. 3b), so a decreased interhemispheric inhibition from LPMC to RPMC may suggest an increased CoV in performance in experts and vice versa in novices.

### Brain–behavior relationships

**Fig. 4:**
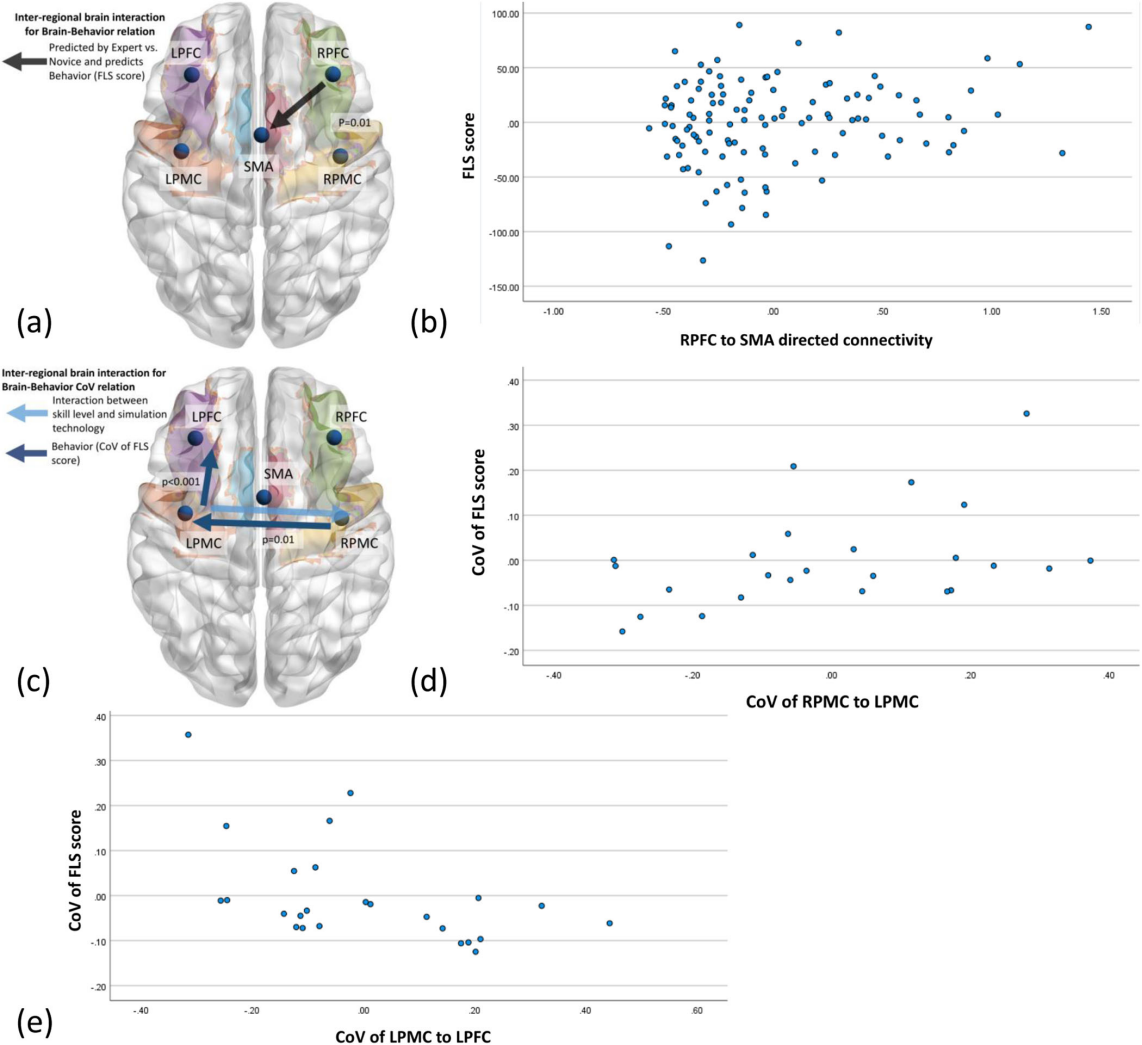
Brain–behavior relationships for the FLS score and the coefficient of variation (CoV) of the FLS score. **a** Interregional directed functional brain connectivity from the RPFC to SMA as a significant (*p* = 0.01) predictor of the FLS score where RPFC to SMA functional connectivity is significantly (*p* < 0.05) affected by skill level (expert vs. novice). **b** The plot shows the partial regression of the interregional directed functional brain connectivity of the RPFC to SMA as a predictor of the FLS score. **c** CoV of the interregional directed functional brain connectivity from the RPMC to LPMC (*p* = 0.01) and LPMC to LPFC (*p* < 0.001) as significant predictors for the CoV of the FLS score. The CoV of the LPMC to RPMC functional connectivity was significantly (*p* < 0.05) affected by the interaction between the skill level and the simulation technology. **d** The plot shows the partial regression of the CoV of interregional directed functional brain connectivity from the RPMC to LPMC as a predictor of the CoV of the FLS score. **e** The plot shows the partial regression of the CoV of the interregional directed functional brain connectivity from the LPMC to LPFC as a predictor of the CoV of the FLS score.

Although the directed functional brain connectivity from the RPFC to SMA, LPMC to SMA, and SMA to LPFC mediated the difference between experts and novices (see Fig. 2), elucidation of the structure of the interaction with the medical simulator technology requires multiple regression and path analysis (MRPA) (SPSS Amos, IBM, USA). For example, the VR simulator was novel for both the expert and the novice, given that experts were experienced with the physical simulator and human surgery. Therefore, an interaction between the skill level and the simulator technology was expected for the interregional directed functional connectivity from the SMA to LPFC, as shown in Fig. 2c, where the directed functional connectivity was similar in the VR simulator for experts and novices. Then, the difference between physical and VR simulators was captured by the directed functional brain connectivity from RPFC to LPMC (see Fig. 2d). Nevertheless, the link between the directed functional brain connectivity and the FLS performance score was missing, so multiple regression (with backward elimination) analysis was performed. The results demonstrate that the FLS score was statistically significantly related to the interregional directed functional connectivity from the RPFC to SMA with *F* (2, 114) = 9, *p* < 0.001, and *R*^2^ = 0.136 (see Fig. 4a). Here, a significant partial regression (*R*^2^ = 0.136) of the dependent variable (FLS score) with the predictor (RPFC to SMA-directed functional connectivity) was found after controlling the false discovery rate of 0.05 with Benjamini–Hochberg adjustment. Supplementary Table 6 presents the ANOVA results. Then, the regression weights from the path analysis (Supplementary Fig. 3) from the factors (expert vs. novice, physical vs. VR simulator) to the directed functional brain connectivity (RPFC to LPMC, RPFC to SMA, LPMC to RPFC, LPMC to SMA, SMA to LPFC) to the FLS performance (FLS score) are shown in Table 1 (grayed rows are significant after controlling for a false discovery rate of 0.05 with Benjamini–Hochberg adjustment).

**Table 1:** Regression weight estimates for path analysis of the FLS score (grayed rows: significant after controlling for a false discovery rate of 0.05 with Benjamini–Hochberg adjustment).

Dependent var.		Independent variable	Weight Estimate	S.E.	C.R.	*P*
RPFC to LPMC	<---	Expert vs. Novice	0.02	0.12	0.13	0.89
RPFC to SMA	<---	Expert vs. Novice	−0.23	0.08	−2.94	0.00
LPMC to RPFC	<---	Expert vs. Novice	0.15	0.11	1.47	0.14
LPMC to SMA	<---	Expert vs. Novice	−0.21	0.09	−2.45	0.01
SMA to LPFC	<---	Expert vs. Novice	0.22	0.09	2.32	0.02
RPFC to LPMC	<---	Physical vs. VR Simulator	0.23	0.12	1.93	0.05
RPFC to SMA	<---	Physical vs. VR Simulator	−0.13	0.08	−1.63	0.10
LPMC to RPFC	<---	Physical vs. VR Simulator	−0.09	0.11	−0.84	0.40
LPMC to SMA	<---	Physical vs. VR Simulator	0.06	0.09	0.64	0.52
SMA to LPFC	<---	Physical vs. VR Simulator	−0.12	0.09	−1.26	0.21
FLS score	<---	RPFC to LPMC	−7.11	5.83	−1.22	0.22
FLS score	<---	RPFC to SMA	21.01	8.43	2.49	0.01
FLS score	<---	LPMC to RPFC	3.66	6.50	0.56	0.57
FLS score	<---	LPMC to SMA	3.13	7.89	0.40	0.69
FLS score	<---	SMA to LPFC	−1.16	7.15	−0.16	0.87

Standard errors (S.E.) and critical ratios (C.R.) are also presented in addition to the *p* value (P) with two significant figures.

When investigating brain–behavior relationships in terms of CoV, multiple regression (with backward elimination) analysis found that the CoV of the FLS score was statistically significantly related to the CoV of the interregional directed functional connectivity from RPMC to LPMC and LPMC to LPFC with *F* (2, 22) = 3.912, *p* = 0.035, and *R*^2^ = 0.262 after controlling for a false discovery rate of 0.05 with Benjamini–Hochberg adjustment (see Fig. 4b). Supplementary Table 7 presents the ANOVA results. Then, the regression weights from the path analysis (Supplementary Fig. 4) from the factors (expert vs. novice, physical vs. VR simulator) to the CoV of the directed functional brain connectivity (RPFC to LPMC, RPFC to SMA, LPMC to RPFC, LPMC to SMA, SMA to LPFC) to the CoV of the FLS performance (FLS CoV) are shown in Table 2 (grayed rows are significant after controlling for a false discovery rate of 0.05 with Benjamini–Hochberg adjustment). Here, the regression weight estimates for the CoV of the RPMC to LPMC was 0.21, and that for the CoV of the LPMC to LPFC was −0.30. Both values were statistically significant after controlling for a false discovery rate of 0.05 with Benjamini–Hochberg adjustment.

**Table 2:** Regression weight estimates for path analysis of CoV of the FLS score (grayed rows: significant after controlling for a false discovery rate of 0.05 with Benjamini–Hochberg adjustment).

Dependent var.		Independent variable	Weight Estimate	S.E.	C.R.	*P*
RPMC to LPMC	<---	Expert vs. Novice	0.05	0.11	0.47	0.64
LPMC to LPFC	<---	Expert vs. Novice	−0.03	0.10	−0.34	0.74
RPMC to LPMC	<---	Physical vs. VR Simulator	0.05	0.11	0.43	0.67
LPMC to LPFC	<---	Physical vs. VR Simulator	−0.02	0.10	−0.19	0.85
LPMC to RPMC	<---	Expert vs. Novice	−0.02	0.13	−0.17	0.87
LPMC to RPMC	<---	Physical vs. VR Simulator	0.08	0.13	0.60	0.55
FLS CoV	<---	RPMC to LPMC	0.21	0.08	2.82	0.01
FLS CoV	<---	LPMC to LPFC	−0.30	0.08	−3.74	<0.001
FLS CoV	<---	LPMC to RPMC	−0.05	0.06	−0.78	0.44

Standard errors (S.E.) and critical ratios (C.R.) are also shown in addition to the *p* value (P) with two significant figures.

## Discussion

Our study established a brain and behavior relationship using MRPA with fNIRS-based directed functional brain connectivity data that showed the feasibility of a portable, low-cost brain-imaging tool to compare task-related cortical information flow in ambulant subjects. Here, validation of medical simulation technology for laparoscopic surgical training based on the brain and behavior relationship is crucial given that psychomotor skill learning or adjusting to changes in the environment, e.g., physical versus VR environment, requires adequate motor exploration, leading to more efficient subsequent learning^13^. In this study, we applied spectral Granger causality^64^ to determine the directional information flow in the brain networks and its CoV in physical and VR simulators. We found that the directed functional brain connectivity (Supplementary Fig. 2) from the RPFC to SMA during FLS task performance mediated the difference between experts and novices and predicted the behavior (FLS score), as shown in Fig. 4a, b. Our results revealed the SMA as the key junction^77^ for the information flow that differentiated the skill level (experts versus novices) (see Fig. 2). Specifically, the SMA region has been considered a key structure^77^ for directed information flow from the LPFC, RPFC, LPMC, and RPMC brain regions during a bimanual sequence operations task^78–81^^,^ as shown in Fig. 1. SMA is a crucial region for interlimb and eye-hand coordination^82–85^ that is critical for perception-action coupling of the temporal organization and bimanual movement execution^78–81^. Therefore, the top-down executive control of the SMA is expected to differ^18^ between experts and novices, where the top-down executive control from PFC^86^ is known to have higher relevance in novices in facilitating training-induced task performance^29^. The directed functional brain connectivity from the RPFC to LPMC differentiated medical simulation technology (physical versus VR simulator) (see Fig. 2), which may be related to different uncertainty in physical versus VR simulators leading to the downstream choice reflected in motor cortex activity^23^.

Additionally, an interaction between medical simulation technology and skill level was captured by the directed functional brain connectivity from the LPMC to the RPFC and the SMA to the LPFC (see Fig. 2), and these findings can be related to the efference copy and collateral discharge, respectively. Here, directed functional brain connectivity from the SMA to the LPFC (see Fig. 2c, e) aligned with our prior work using wavelet coherence-based functional connectivity measures^87^ that found undirected functional connectivity between the PFC and SMA to be lower for experts than novices in the physical simulators. Therefore, a directed functional connectivity approach^62^ to the fNIRS time series could capture the cascading directional processing of goal-directed action^69^, as postulated based on the dorsal stream of action in Fig. 1b. In this study, sliding-window Granger causality provided a tool for identifying directed functional interactions from the fNIRS time series data that did not assume a static functional brain network across the whole FLS task. Thus, this method could also capture the CoV across repeated trials of the FLS task. An interaction effect between the skill level and the simulator technology for the CoV was found for the directed functional brain connectivity from the LPMC to RPMC, as shown in Fig. 3. This finding aligned with our prior work using a wavelet coherence-based functional brain connectivity measure^88^ that elucidated the brain–behavior relationship based on the CoV between the LPMC-RPMC magnitude-squared wavelet coherence metric and the FLS score; however, the directionality of the information flow was not investigated earlier. Then, path analysis for the brain–behavior relation showed that the CoV of the directed functional brain connectivity from the RPMC to LPMC and LPMC to LPFC were significant predictors for the CoV of the FLS score, as shown in Fig. 4c–e. Here, our current study highlighted the importance of portable brain imaging to evaluate medical simulation technology. Specifically, Granger causality and a multiple regression approach identified the directed information flow related to efference copy and corollary discharge linked to predictive internal signaling^89^ that mediated the interaction between skill level and medical simulation technology.

In this study, we found that portable brain imaging for brain–behavior modeling can evaluate medical simulation technology in terms of its interaction with the skill level within the context of the perception-action cycle^26^. This difference was captured by the directed functional brain connectivity from the RPFC to LPMC (Fig. 2) during FLS task performance, which was higher in the VR simulator than in the physical simulator for both experts and novices. Here, the PFC is postulated to subserve cognitive control^90^ and attentional processes^76^ that can depend on the uncertainty^23^ underlying FLS training with different medical simulation technologies. Then, the distinguishing directed information flow for skill level as a predictor of FLS performance was found to proceed from the RPFC to SMA (see Fig. 4a), which trended toward being lower in the VR simulator than in the physical simulator (see Fig. 2b). Jenkins et al.^91^ demonstrated that PFC activation is associated with the learning of new sequence tasks, whereas the lateral premotor cortex is more activated during new learning and the SMA is more activated during the performance of a prelearned sequence. Therefore, the descent of the information flow from the PFC to the premotor/motor cortex^92,93^ is expected in a VR simulator that was novel for both the expert and the novice and affected the exploration strategy^94^. Specifically, experts had prior knowledge of the FLS task and thus could have used directed exploration in the VR simulator, whereas novices could have depended on random exploration in the initial stages of the FLS task^23^ in the VR simulator. This investigation of different exploration strategies will require a higher density fNIRS optode montage to segregate the dorsolateral, ventrolateral, and rostrolateral PFC^23^ in our future work. The dorsolateral and ventrolateral PFC can be related to attention control, cognitive control, feature extraction, and the formation of first-order relationships^45,95–97^ that are relevant during the initial stage of motor skill learning in novices. Specifically, the dorsolateral PFC of the dorsal stream is more involved in the visual guidance of action, whereas the ventrolateral PFC of the ventral stream is more involved in recognition and conscious perception^98^. Then, the SMA and the PMC are crucial for coordinating bimanual movement^99^^,^ where SMA is crucial for the complex spatiotemporal sequencing of movements^79,100^ necessary in FLS tasks. Then, in the later stage of motor skill learning for proficiency^16^, rostrolateral PFC may drive directed exploration based on relative uncertainty^23^ to improve the robustness of the internal models.

In this study, an interaction between the medical simulation technology (physical vs. VR simulator) and the skill level (experts vs. novices) was found from the directed functional brain connectivity from the LPMC to the RPFC and the SMA to the LPFC (see Fig. 2f, e), and this interaction can be related to efference copy and corollary discharge information flow, respectively (see Fig. 1b). Here, the SMA contributes to the prediction of the sensory consequences of the sequence of subtask-related movement^101^, which is expected when an internal forward model is available for fine motor control (e.g., for experts in the physical simulator). Therefore, corollary discharge^102^ from the SMA to the PFC is expected for experts who have experienced physical simulators and human surgery for bimanual complex movement^79,100^. However, the VR simulator was novel for both the experts and the novices, so the corollary discharge^102^ from the SMA to the PFC was reduced from the physical to VR simulator in the experts and was comparable to the novices in the VR simulator (see Fig. 2e). Furthermore, the efference copy from the LPMC to the RPFC is postulated to be related to the functional coupling of the prefrontal and premotor/motor areas that are expected during cognitive manipulation^103^ under uncertain conditions^23^. Here, an increased cognitive manipulation^103^ under higher uncertainty^23^ for both the experts and the novices (both inexperienced in VR) is postulated in the VR simulator compared to the physical simulator^70^, i.e., an increased information flow from the RPFC to the LPMC in the VR simulator (see Fig. 2d). Additionally, the efference copy from the LPMC to the RPFC was reduced from the physical simulator to the VR simulator in the experts due to a lack of an internal model such that the LPMC- to RPFC-directed functional connectivity in the experts was comparable to that in the novices in the VR simulator (see Fig. 2f).

Our prior work^65^ established the face and construct validity of the VR simulator that is consistent with the current study results, where only the skill level and not the simulator technology exhibited a significant effect on the FLS score and its CoV (see Fig. 3a, b). Specifically, the expert had a higher FLS score (Fig. 3a) and lower CoV (Fig. 3b) than the novice in the physical simulator; however, in the VR simulator, the expert without VR experience trended toward a similar level as the novice. Here, motor variability influencing task performance has been postulated to shape motor learning^104,105^, and motor variability typically tends to decrease with practice^106^, which tends to drive the trade-off between exploitation and exploration^107^. Subjects are expected to learn to avoid the influence of motor variability on goal-directed task performance^104,105^, as observed in the experts with reduced CoV in the task performance (FLS score) in the physical simulator than novices. Both experts and novices exhibited similar CoV in the novel VR simulator (Fig. 3b). The variability (CoV) in the task performance (FLS score) was significantly related to the variability (CoV) in the directed functional brain connectivity from the RPMC to LPMC and LPMC to LPFC, as shown in Fig. 4c, d, and 4e, which presented a neural correlate of performance variability^108^. Here, an increase in the CoV of the RPMC to LPMC and a decrease in the CoV of LPMC to LPFC were related to an increase in the CoV of the FLS score.

Additionally, an effect of the interaction between the skill level and the simulator technology was found on the CoV of the directed functional brain connectivity from the LPMC to RPMC, as shown in Fig. 3c. The efference copy information from the bilateral motor cortices to the LPFC highlighted the left-lateralized perceptual decision-making associated with behavioral variability (FLS score). Here, we found hemispheric lateralization in perception-action coupling in our right-handed subjects where the coupling between the LPMC and the RPFC (see Fig. 2d, f) can be related to sensory prediction (efferent copy from LPMC to RPFC) for action (executive control from RPFC to LPMC) to respond to unexpected environmental stimuli^109,110^ in the VR environment. Additionally, uncertainty due to unexpected environmental stimuli^109^ is subserved by the right PFC, where relative uncertainty (e.g., in experts) is represented in the right rostrolateral PFC, whereas total uncertainty (e.g., in novices) is represented in the right dorsolateral PFC^23^. In contrast, the involvement of LPFC (see Fig. 2) as the recipient of the corollary discharge information from SMA for action-perception coupling may be related to its role in analyzing external information while planning a goal hierarchy^110^ for proficient subtask sequencing. Then, any conflict between the efferent information and the sensory reafferent information can lead to a loss of subjective sense of agency in the angular gyrus^72^ (see Fig. 1b). Future work on improving the design of the VR simulator needs to address the brain–behavior relationship by reducing the conflict between the efferent information and the sensory reafferent information to facilitate the sense of agency associated with learnability^111^ and eventually motor skill “automaticity”^18^. Specifically, brain–behavior monitoring can be used to drive the virtual environment in ‘real-time’ to calibrate according to the degree of adaptation of the user’s prediction models to create a subjective sense of agency in novices as they learn psychomotor tasks with increasing task complexity, i.e., an adaptive VR simulator.

The experimental results of this study are conducive to the exploration of transcranial electrical stimulation^112^ to facilitate learnability in medical simulators. For example, mobile brain–behavior analysis with fNIRS can capture the interaction between the angular gyrus (AG) and the middle frontal gyrus (MFG) that is underpinned by the dorsal superior longitudinal fascicle (SLF II)^113^, and the subjective sense of agency may be facilitated by neuroimaging-guided transcranial electrical stimulation^112^ of the AG-MFG interactions^114^. The dorsal branch of the superior longitudinal fasciculus, which is responsible for visuospatial integration and motor planning, is linked to lateralized hand preference and manual specialization^115^. Here, the right MFG has been proposed to be a site of convergence of the dorsal and ventral attention networks^76^ for cognitive control that is relevant in the perception-action cycle. The ventral superior longitudinal fascicle (SLF III)^113^ is postulated to be more relevant in perception (see Fig. 1b) from the supramarginal gyrus (SMG), where the left MFG and left inferior frontal gyrus (IFG) are more involved in more perceptually demanding FLS tasks, e.g., FLS suturing with intracorporal knot tying^116^. Here, the ventral stream of perception can be facilitated by neuroimaging-guided transcranial electrical stimulation^112^ of SMG-IFG interactions^114^. Then, the coupling between the SMA and LPFC may be related to patterns of prelearned sequence of motor behavior performed in familiar environments^109^ in the case of experts in the physical simulator. Here, it is postulated that the interaction between the preSMA/SMA and the PFC/IFG is underpinned by the extended frontal aslant tract (exFAT)^117^ of the short frontal lobe connections^118^ that have a role in executive function/ability^119^. The exFAT may be left-lateralized^117^, which aligns well with left-lateralized activation for more complex bimanual FLS tasks, e.g., FLS suturing with intracorporal knot tying^116^. So, transcranial electrical stimulation^112^ may facilitate the development of internal models^120^ as well as efference copy and corollary discharge information flows, which may facilitate predictive internal signaling^89^.

Limitations of this study include the spatial resolution of fNIRS and the optimality of the parameter of the sliding-window method for measuring dynamic functional connectivity^62^. The smallest window greater than 50 sec was found by running stationarity tests on the fNIRS time series. Here, a trade-off was made, i.e., on the one hand, the window must be long enough to provide good frequency resolution, and on the other hand, the window must be short enough to satisfy the condition of stationarity. Therefore, instead of an ad hoc window size^121^, we searched for an optimal^122^ sliding-window pertinent to our data. In this study, we investigated the first sliding window of 54 s across five repeated trials of FLS tasks when the cutting was performed with the right hand for all right-handed subjects (the cutting direction and the hand switched at different timepoints after 54 s due to the surgical field constraints; see the FLS pattern cutting video in the Supplementary Materials). Therefore, we aimed to capture the initial stage in FLS pattern cutting skill acquisition to investigate the action-perception link^70^ when the perceptual model^7^ is being developed. Then, due to the limitations of the spatial resolution of our fNIRS device, we investigated only five brain regions, including the LPFC, RPFC, LPMC, RPMC, and SMA. Here, the premotor and motor areas were combined in the PMC (see Supplementary Table 1), and the fNIRS optode montage could not distinguish the SMA proper from the preSMA brain regions, which may be important to better assess the temporal structure^123^ of the perception-action coupling link^70^. Additionally, we did not investigate all of the subregions of the PFC, e.g., the ventrolateral PFC and inferior frontal gyrus (IFG), that may have essential functional interactions during FLS surgical skill acquisition^112^, where the feasibility of fNIRS’ temporal resolution needs to be demonstrated in the future to capture the fast interactions that are expected via shorter frontal lobe connections^118^.

## Methods

### Subjects and experimental design

The human study was approved by the Institutional Review Board of the Massachusetts General Hospital, University at Buffalo, and the Rensselaer Polytechnic Institute, USA. Convenience sampling recruited seven experienced right-handed surgeons (experts, 5th-year residents, and attending surgeons) and six right-handed medical students (novices, 1st- to 3rd-year residents) to participate in the study. The subject details are provided in Table 3. Only right-handed subjects were selected to avoid dominant hemisphere-related intersubject variability.

**Table 3:** Subject demographics.

Subject	Age	Gender	Specialization
**Physical simulator**			
Novice			
N1	32 years	Male	General Surgery
N2	31 years	Female	General Surgery
N3	30 years	Male	General Surgery
N4	33 years	Male	General Surgery
N5	32 years	Female	General Surgery
N6	30 years	Male	Orthopedic Surgery
Expert			
E1	38 years	Female	General Surgery
E2	49 years	Female	General Surgery
E3	30 years	Male	General Surgery
E4	30 years	Female	General Surgery
E5	37 years	Male	General Surgery
E6	32 years	Female	General Surgery
E7	31 years	Male	General Surgery
**VR simulator**			
Novice			
N1	31 years	Female	General Surgery
N2	30 years	Male	General Surgery
N3	33 years	Male	General Surgery
N4	32 years	Female	General Surgery
N5	30 years	Male	Orthopedic Surgery
N6	26 years	Male	General Surgery
Expert			
E1	38 years	Female	General Surgery
E2	49 years	Female	General Surgery
E3	30 years	Male	General Surgery
E4	30 years	Female	General Surgery
E5	37 years	Male	General Surgery
E6	34 years	Male	General Surgery

Written consent was obtained from each subject before starting the study. All subjects were instructed verbally with a standard set of instructions on how to complete the FLS pattern cutting task on the FLS-certified physical and the VR simulator^124^. For the completion of the FLS pattern cutting task, the right-handed subjects were asked to grasp the gauze using the left grasper (for traction) and cut along (and within) the circular stamp with the right laparoscopic scissors (for cutting). The trial time started when the subject touched the gauge and ended when the circular cut piece was removed from the gauge frame, and the participants were asked to cut the marked piece of gauze as quickly and as accurately as possible. Data collection was performed with a block design of a rest and stimulus period (the pattern cutting task). Specifically, after a 1-min rest period (baseline data), the FLS pattern cutting task had to be completed or stopped within 5 min (task data). This was repeated five times (5 trials) for each participant in this repeated-measure study. The performance score for each trial was recorded based on the FLS metrics.

A 32-channel continuous-wave near-infrared spectrometer (CW6 system, TechEn Inc., USA) was used for optical brain imaging using infrared light at 690 and 830 nm. The optode montage consisted of eight long-distance and eight short-distance sources coupled with 16 detectors. Twenty-five long-distance (30–40 mm) channels and eight short-distance (~8 mm) channels measured brain activation and systemic physiological signals, respectively (brain regions listed in Supplementary Table 1) that were assessed using the photon migration simulation in AtlasViewer software^125^. Here, the photon migration forward matrix represents the sensitivity profile. We selected the average fNIRS signal of the left and right middle frontal gyrus for prefrontal cortex activation, i.e., LPFC and RPFC; the left and right precentral gyrus for premotor/motor cortex activation, i.e., LPMC and RPMC; and the bilateral supplementary motor area complex for supplementary motor area activation, i.e., SMA. Supplementary Table 1 provides the Montreal Neurological Institute and Hospital (MNI) coordinates. The optical fibers were duly arranged in a cap so that they did not obstruct the free movement of the participant during the FLS task performance.

### fNIRS data processing for the oxyhemoglobin time series

Motion artifact detection and correction were performed using Savitzky–Golay filtering^126^ and bandpass filtering (0.01–0.1 Hz) in HOMER3 software (https://github.com/BUNPC/Homer3). Then, the modified Beer–Lambert law was used to convert the optical signals of the detectors into changes in the oxyhemoglobin (HbO_2_) concentrations for partial path-length factors of 6.4 (690 nm) and 5.8 (830 nm). The short separation channels (interoptode distance of 8 mm) captured the systemic physiological signals originating from noncortical superficial regions. The averaged signal from the long separation channels (interoptode distance of 30–40 mm) measured the HbO_2_ changes at the following brain regions: LPFC, RPFC, LPMC, RPMC, and SMA. Supplementary Fig. 5 shows an illustrative plot of the HbO_2_ time series.

### Granger causality analysis

Granger causality using custom code measured the directed functional connectivity that provided the strength and direction of cortical information flow^67^ between a pair of brain regions from the LPFC, RPFC, LPMC, RPMC, and SMA^64^. Granger causality is grounded upon the postulate that one “causally” connected region would leave a component of its signal on another region with some latency, i.e., an autoregressive model (Granger Causality Description provided in the Supplementary Materials)^127,128^. Here, short-time Fourier transformation (STFT) was employed for nonparametric spectral Granger causality to estimate sliding-window pairwise measures of Granger causality, thereby eliminating the need for explicit autoregressive modeling^67^. The lowest frequency of 0.02 Hz was found from the fNIRS power spectral density (after 0.01–0.1 Hz bandpass filtering), so a nonoverlapping fixed window size of 54 sec (greater than 50 sec) was selected heuristically^129^.

### Directed functional brain network

Granger causality in the neurovascular frequency range of 0.01 to 0.07 Hz^87^ was used to obtain directed connectivity for each pair of regions (a total of 20 connections). Supplementary Fig. 1 shows an example of 20 interregional directed connections for an illustrative time window. The directed connectivity between each pair of brain regions was used to form the directed functional brain network at each time window. Here, a neurovascular frequency range of 0.01 to 0.07 Hz^87^ acted as a filter for systemic and physiological noise, such as heartbeat and respiration^130^.

### Statistical analysis

Individual data collected from seven experienced right-handed surgeons and six right-handed medical students during the performance of FLS pattern cutting tasks in a physical simulator and a VR stimulator were used to determine the Granger causal directed functional connectivity metric for each subject (seven experts, six novices) with each simulator technology (physical, VR). The Shapiro–Wilk test was used to test normality for each of the dependent variables (i.e., interregional directed functional connectivity metric). Supplementary Table 8 shows the results from Shapiro–Wilk’s test of normality for the Granger causality measure of each pair of brain regions in experts and novices while performing FLS tasks in the physical and virtual simulators across five trials. Then, the directed functional connectivity (Granger causality) between each pair of brain regions for the first window (54 s) of each trial was used to conduct a repeated-measure two-way MANOVA in SPSS version 27 (IBM, USA) to determine whether there was a significant difference in the interregional directed functional connectivity based on the skill level (expert, novice), simulator technology (physical simulator, VR simulator) and their interaction. We conducted two-way MANOVA in SPSS version 27 (IBM, USA) to determine whether a significant difference in the CoV of interregional directed functional connectivity was noted across trials based on skill level, simulator technology, and their interaction. We conducted a repeated-measure two-way ANOVA in SPSS version 27 (IBM, USA) to determine whether there was a significant difference in the FLS score based on the skill level, simulator technology, and their interaction. We also conducted a repeated-measure two-way ANOVA in SPSS version 27 (IBM, USA) to determine whether there was a significant difference in the CoV of the FLS score based on skill level, simulator technology, and their interaction. The Levene test was used to test the homogeneity of variance. All significance levels were set at alpha = 0.05. To determine how the dependent variables (i.e., interregional directed functional connectivity) differed for the independent variables, the skill level, simulator technology, and their interaction, i.e., the tests of between-subjects effects, alpha with multiple comparison correction (false discovery rate), and partial eta squared effect size were used. Here, partial eta squared effect size measures the proportion of the total variance in a dependent variable defined by an independent variable in which the effects of other independent variables and interactions are parsed out^131^. Then, correction for a false discovery rate of 0.05 with Benjamini–Hochberg adjustment was performed using the MATLAB code in the Supplementary Materials. Then, we conducted brain–behavior analysis via multiple regression (backward elimination with a probability of F for removal ≥0.1) in SPSS version 27 (IBM, USA) to assess the relationship of the interregional directed functional brain connectivity with the FLS score. Then, in SPSS Amos (IBM, USA), the path analysis was performed from the skill level (expert, novice) and simulator technology (physical simulator, VR simulator) to the dependent variables (interregional directed functional brain connectivity and FLS score). Multiplicity control for the path analysis was also based on a false discovery rate of 0.05 with Benjamini–Hochberg adjustment using the MATLAB code in the Supplementary Materials.

### Reporting summary

Further information on research design is available in the Nature Research Reporting Summary linked to this article.

## Supplementary information

FLS pattern cutting task video

Supplementary Materials

Reporting Summary Checklist

## Data Availability

All processed data analyzed during this study are included in this published article (and its Supplementary Information files).

## Code availability

The code generated during the current study is publicly available at https://github.com/anilkamat/GC_FLS_PC.git.
