# Supplementary material for: Directed information flow during laparoscopic surgical skill acquisition dissociated skill level and medical simulation technology
Source: NPJ Sci Learn. 2022 Aug 25;7:19. doi: 10.1038/s41539-022-00138-7 (PMC9411170; doi:10.1038/s41539-022-00138-7)
Supplement: Supplementary file 2 — Supplementary Materials [file 41539_2022_138_MOESM2_ESM.pdf]

Supplementary Materials

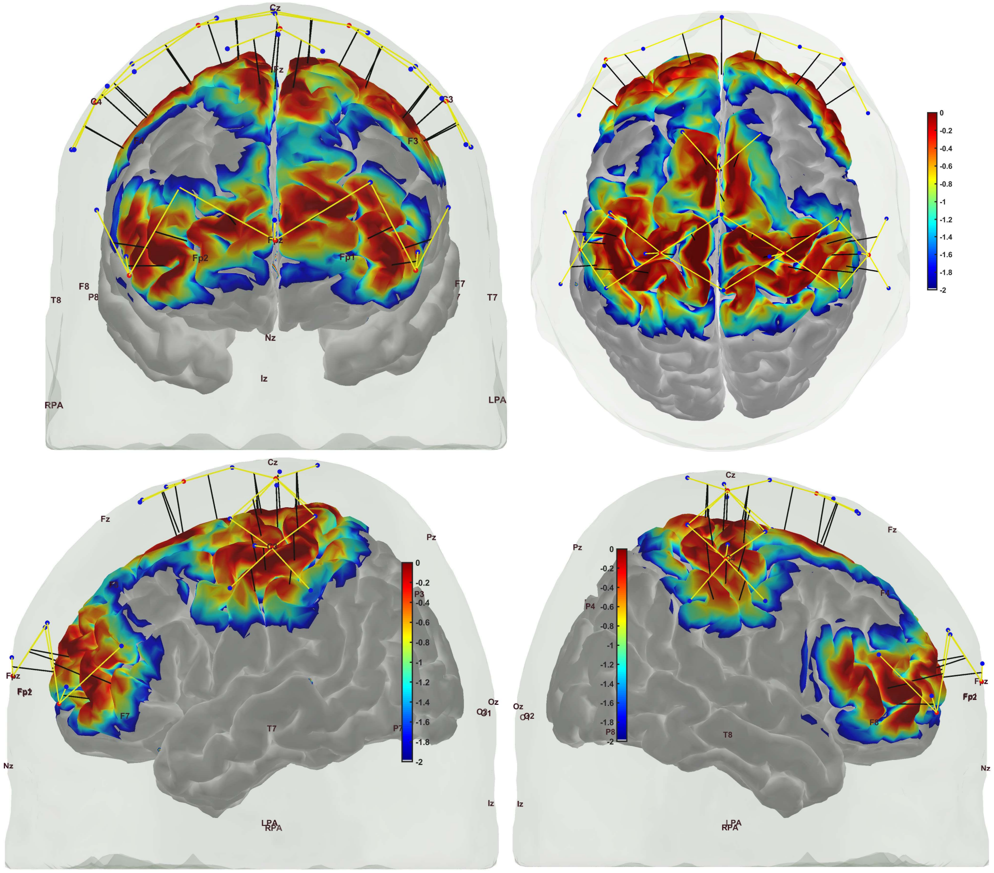

Supplementary Figure 1: *Sensitivity profile of the optode montage covering the left and right middle frontal gyrus for prefrontal cortex activation, the left and right precentral gyrus for premotor/motor cortex activation, and the bilateral supplementary motor area complex for supplementary motor area activation. The color scale depicts the sensitivity logarithmically from 0.01 to 1.*

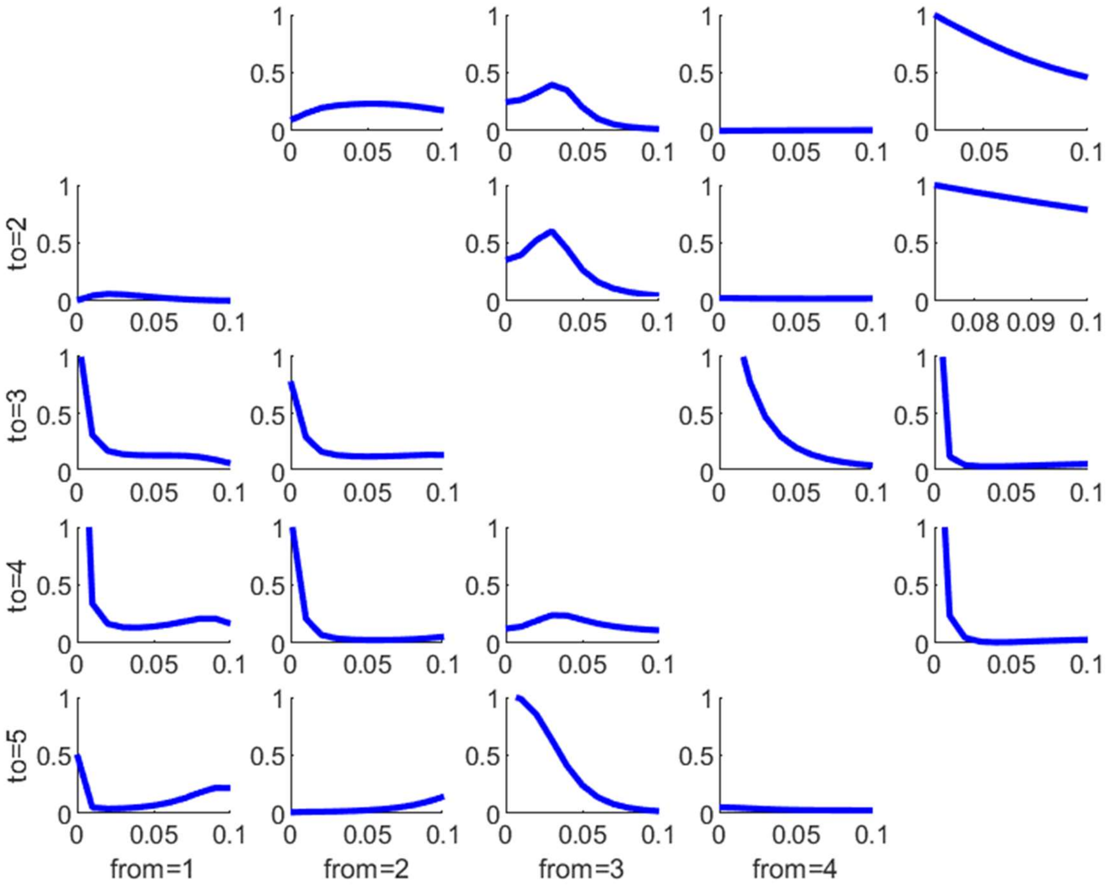

Supplementary Figure 2: *Illustrative example of bidirectional connectivity in one of the windows obtained using the short-time Fourier transformation (STFT) method. Each subplot represents the strength of the association from one brain region to another for a band of frequency, where the horizontal axis is the frequency, and the vertical axis is the Granger causality. Link numbers 1, 2, 3, 4 and 5 represent the LPFC, RPFC, SMA, LPMC, and RPMC, respectively. The directed functional connectivity is shown for frequencies from 0 Hz to 0.1 Hz within the neurovascular coupling band; however, the directed functional connectivity in the range of ultralow frequency,* i.e.*, 0.01 Hz to 0.07 Hz, was obtained for the analysis.*

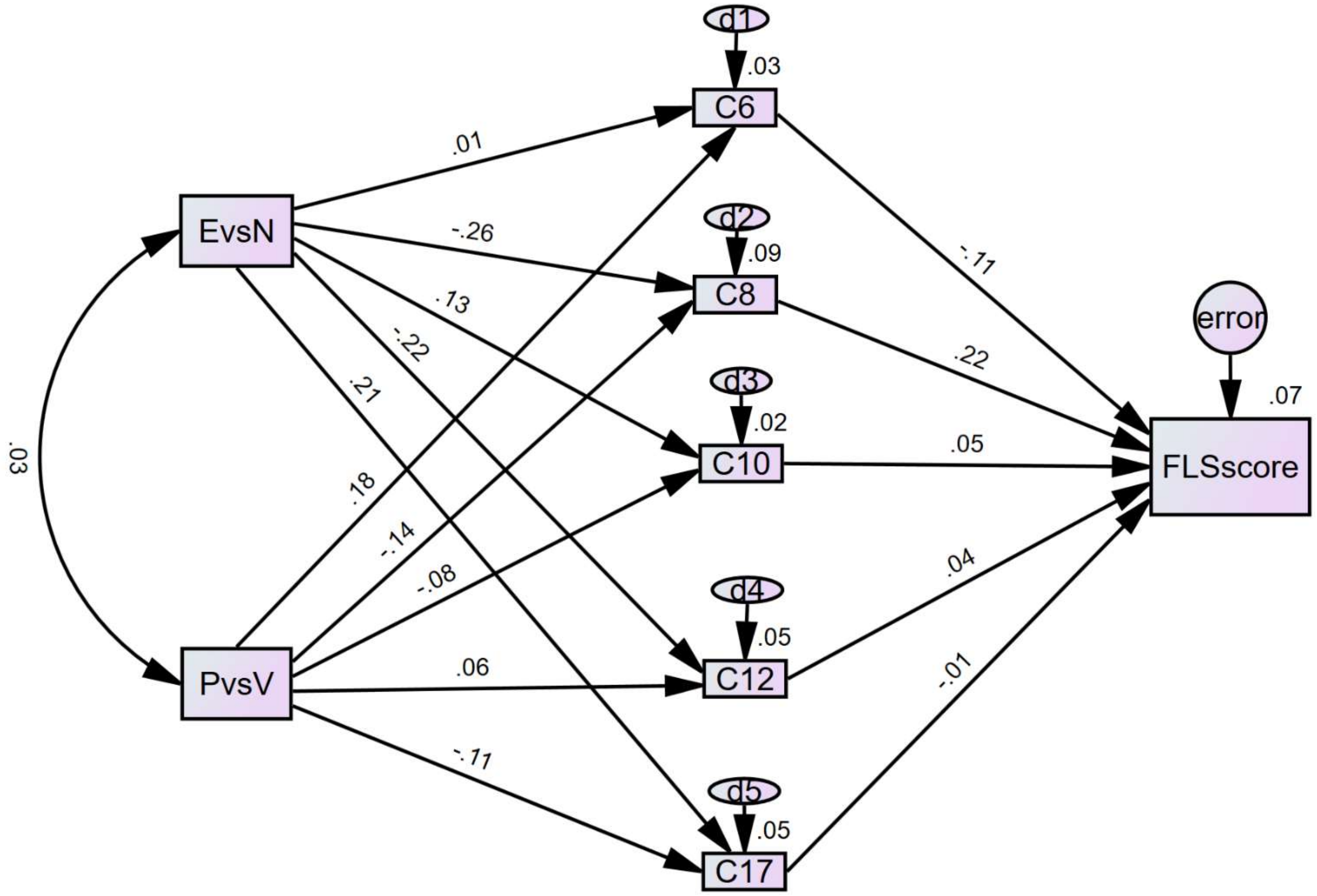

Supplementary Figure 3: *Path analysis from factors (expert* vs. *novice: EvsN, physical* vs. *VR simulator: PvsV) to the directed functional brain connectivity (C6: RPFC to LPMC, C8: RPFC to SMA, C10: LPMC to RPFC, C12: LPMC to SMA, C17: SMA to LPFC) to the FLS performance score (FLSscore). Here, d1, d2, d3, d4, and d5 are disturbances or noise in the directed functional brain connectivity, and a separate 'error' occurs when predicting the FLSscore.*

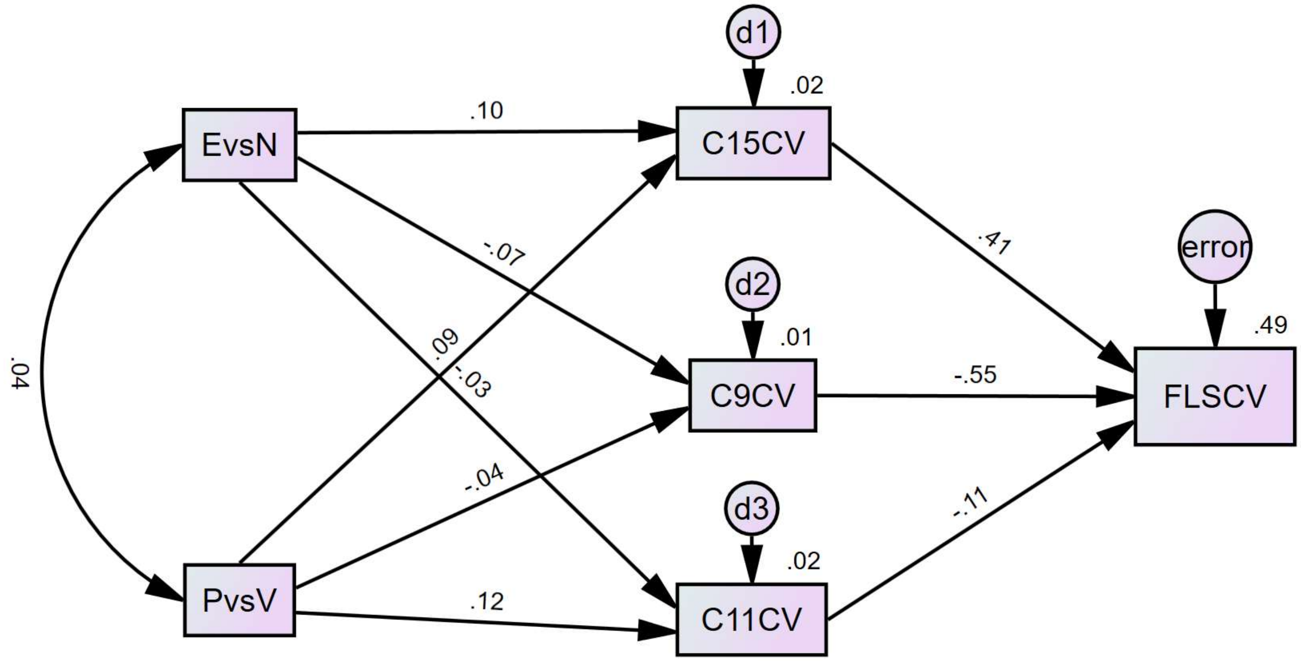

Supplementary Figure 4: *Path analysis from factors (expert* vs. *novice: EvsN, physical* vs. *VR simulator: PvsV) to the coefficient of variation (CoV) of directed functional brain connectivity (C15CV: RPMC to LPMC, C9CV: LPMC to LPFC, C11CV: RPMC to LPMC) to the CoV FLS performance score (FLSCV). Here, d1, d2, and d3 are disturbances or noise in the directed functional brain connectivity, and a separate 'error' occurs when predicting FLSCV.*

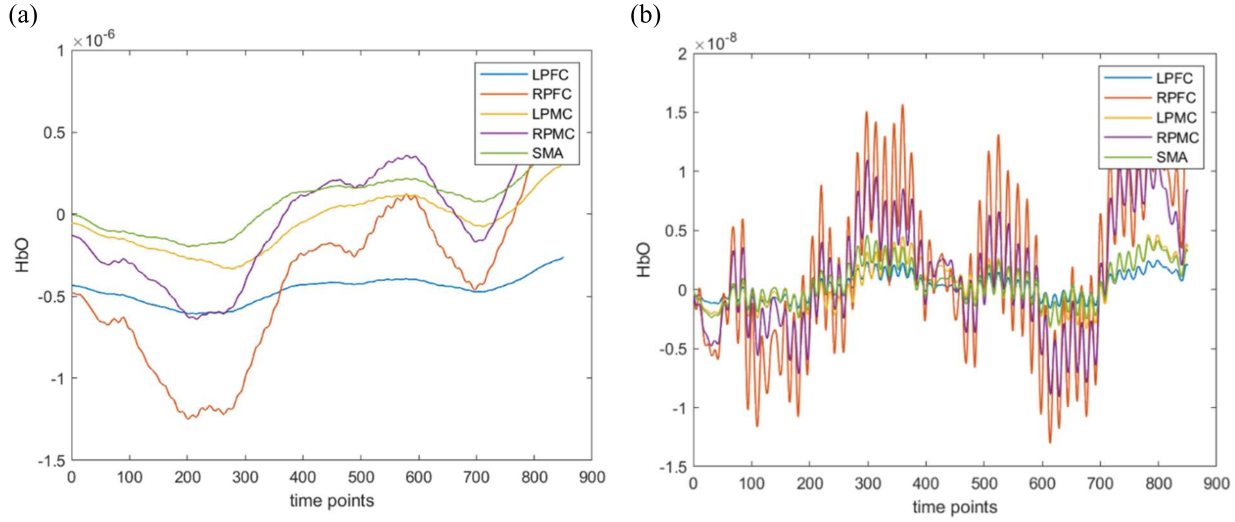

Supplementary Figure 5: *(a) Representative of hemodynamic changes of oxyhemoglobin signal (HbO_2) from the five independent regions of interest in the one window. (b) Signals after integration of degree one I(1) converges to zero mean and variance.*

Supplementary Table 1: *Brain regions targeted with fNIRS based on source (SRC) detector (DET) coupling and automated anatomical labeling (AAL) of those brain regions with Montreal Neurological Institute (MNI) coordinates.*

LABEL	SRC	DET	MC COORD	MNI COORD	AAL LABEL NAME
LPFC	1	1	82 143 188	50 46 7	Frontal_Mid_R
LPFC	1	2	92 137 205	40 63 13	Frontal_Mid_R
	1	17	88 149 194	44 52 1	Frontal_Mid_Orb_R
	2	2	115 130 208	17 66 20	Frontal_Sup_Medial_R
	2	3	140 138 195	-8 53 12	Frontal_Sup_Medial_L
	2	18	129 137 207	3 65 13	Frontal_Sup_Medial_L
RPFC	3	3	164 138 201	-32 59 12	Frontal_Mid_L
RPFC	3	4	159 144 178	-27 36 6	Frontal_Mid_L
	3	19	164 148 187	-32 45 2	Frontal_Mid_L
LPMC	4	5	76 102 129	56-13 48	Precentral_R
LPMC	4	6	76 102 115	56-27 48	Precentral_R
LPMC	4	7	83 95 127	49-15 55	Precentral_R
LPMC	4	8	77 89 112	55-30 61	Precentral_R
	4	20	90 108 123	42-19 42	Precentral_R
	5	7	95 82 125	37-17 68	Precentral_R
	5	8	103 95 109	29-33 55	Postcentral_R
	5	9	117 77 125	15-17 73	Precentral_R
	5	10	116 77 111	16-31 73	Precentral_R
	5	21	106 74 119	26-23 76	Postcentral_R
	6	9	143 75 127	-11-15 75	Paracentral_Lobule_L
	6	10	143 66 110	-11-32 84	Postcentral_L
	6	11	152 91 126	-20-16 59	Precentral_L
	6	12	154 88 115	-22-27 62	Postcentral_L
	6	22	149 72 118	-17-24 78	Postcentral_L
RPMC	7	11	163 106 120	-31-22 44	Precentral_L
RPMC	7	12	161 106 114	-29-28 44	Precentral_L
RPMC	7	13	187 103 125	-55-17 47	Precentral_L
RPMC	7	14	170 112 117	-38-25 38	Precentral_L
	7	23	176 100 119	-44-23 50	Precentral_L
SMA	8	9	130 71 145	2 3 79	Supp_Motor_Area_L
SMA	8	15	123 85 161	9 19 65	Supp_Motor_Area_R
SMA	8	16	135 76 162	-3 20 74	Supp_Motor_Area_L
	8	24	129 78 160	3 18 72	Supp_Motor_Area_L

Supplementary Table 2: *Tests of between-subjects effects.*

Transformed Variable: Average

Source	Measure	Type III Sum of Squares	df	Mean Square	F	Sig.	Partial Eta Squared	Noncent. Parameter	Observed Power[a]
Intercept	LPFC to RPFC	60.078	1	60.078	198.366	0.000	0.930	198.366	1.000
	LPFC to LPMC	61.989	1	61.989	176.451	0.000	0.922	176.451	1.000
	LPFC to RPMC	42.486	1	42.486	69.330	0.000	0.822	69.330	1.000
	LPFC to SMA	25.323	1	25.323	90.101	0.000	0.857	90.101	1.000
	RPFC to LPFC	45.761	1	45.761	90.580	0.000	0.858	90.580	1.000
	RPFC to LPMC	59.146	1	59.146	151.383	0.000	0.910	151.383	1.000
	RPFC to RPMC	53.532	1	53.532	151.835	0.000	0.910	151.835	1.000
	RPFC to SMA	21.911	1	21.911	81.401	0.000	0.844	81.401	1.000
	LPMC to LPFC	39.515	1	39.515	102.755	0.000	0.873	102.755	1.000
	LPMC to RPFC	45.689	1	45.689	182.656	0.000	0.924	182.656	1.000
	LPMC to RPMC	40.698	1	40.698	64.863	0.000	0.812	64.863	1.000
	LPMC to SMA	20.553	1	20.553	62.769	0.000	0.807	62.769	1.000
	RPMC to LPFC	37.151	1	37.151	179.762	0.000	0.923	179.762	1.000

	RPMC to RPFC	33.203	1	33.203	152.881	0.000	0.911	152.881	1.000
	RPMC to LPMC	40.556	1	40.556	74.431	0.000	0.832	74.431	1.000
	RPMC to SMA	41.193	1	41.193	122.836	0.000	0.891	122.836	1.000
	SMA to LPFC	35.979	1	35.979	142.487	0.000	0.905	142.487	1.000
	SMA to RPFC	37.334	1	37.334	150.008	0.000	0.909	150.008	1.000
	SMA to LPMC	36.562	1	36.562	99.162	0.000	0.869	99.162	1.000
	SMA to RPMC	33.476	1	33.476	201.028	0.000	0.931	201.028	1.000
EvsN	LPFC to RPFC	0.184	1	0.184	0.607	0.448	0.039	0.607	0.113
	LPFC to LPMC	0.151	1	0.151	0.430	0.522	0.028	0.430	0.094
	LPFC to RPMC	0.026	1	0.026	0.042	0.840	0.003	0.042	0.054
	LPFC to SMA	1.051	1	1.051	3.741	0.072	0.200	3.741	0.441
	RPFC to LPFC	0.013	1	0.013	0.026	0.873	0.002	0.026	0.053
	RPFC to LPMC	0.001	1	0.001	0.002	0.968	0.000	0.002	0.050
	RPFC to RPMC	0.082	1	0.082	0.231	0.637	0.015	0.231	0.074
	RPFC to SMA	1.627	1	1.627	6.045	0.027	0.287	6.045	0.633

	LPMC to LPFC	0.039	1	0.039	0.101	0.755	0.007	0.101	0.060
	LPMC to RPFC	1.132	1	1.132	4.526	0.050	0.232	4.526	0.512
	LPMC to RPMC	0.077	1	0.077	0.123	0.731	0.008	0.123	0.062
	LPMC to SMA	2.584	1	2.584	7.892	0.013	0.345	7.892	0.747
	RPMC to LPFC	0.057	1	0.057	0.278	0.606	0.018	0.278	0.078
	RPMC to RPFC	0.085	1	0.085	0.389	0.542	0.025	0.389	0.090
	RPMC to LPMC	0.069	1	0.069	0.126	0.727	0.008	0.126	0.063
	RPMC to SMA	0.092	1	0.092	0.275	0.608	0.018	0.275	0.078
	SMA to LPFC	1.664	1	1.664	6.591	0.021	0.305	6.591	0.670
	SMA to RPFC	0.510	1	0.510	2.047	0.173	0.120	2.047	0.268
	SMA to LPMC	0.174	1	0.174	0.472	0.503	0.030	0.472	0.099
	SMA to RPMC	0.010	1	0.010	0.063	0.805	0.004	0.063	0.056
PvsV	LPFC to RPFC	0.088	1	0.088	0.290	0.598	0.019	0.290	0.080
	LPFC to LPMC	0.286	1	0.286	0.813	0.381	0.051	0.813	0.135
	LPFC to RPMC	0.002	1	0.002	0.003	0.961	0.000	0.003	0.050

	LPFC to SMA	0.219	1	0.219	0.778	0.392	0.049	0.778	0.131
	RPFC to LPFC	0.476	1	0.476	0.942	0.347	0.059	0.942	0.149
	RPFC to LPMC	2.345	1	2.345	6.002	0.027	0.286	6.002	0.630
	RPFC to RPMC	0.014	1	0.014	0.039	0.845	0.003	0.039	0.054
	RPFC to SMA	0.418	1	0.418	1.552	0.232	0.094	1.552	0.215
	LPMC to LPFC	0.298	1	0.298	0.775	0.393	0.049	0.775	0.131
	LPMC to RPFC	0.731	1	0.731	2.922	0.108	0.163	2.922	0.360
	LPMC to RPMC	0.116	1	0.116	0.185	0.673	0.012	0.185	0.069
	LPMC to SMA	0.464	1	0.464	1.417	0.252	0.086	1.417	0.200
	RPMC to LPFC	0.010	1	0.010	0.050	0.826	0.003	0.050	0.055
	RPMC to RPFC	0.391	1	0.391	1.801	0.200	0.107	1.801	0.242
	RPMC to LPMC	0.068	1	0.068	0.125	0.729	0.008	0.125	0.063
	RPMC to SMA	0.007	1	0.007	0.020	0.890	0.001	0.020	0.052
	SMA to LPFC	0.954	1	0.954	3.778	0.071	0.201	3.778	0.444
	SMA to RPFC	0.033	1	0.033	0.131	0.722	0.009	0.131	0.063

	SMA to LPMC	0.216	1	0.216	0.586	0.456	0.038	0.586	0.111
	SMA to RPMC	0.019	1	0.019	0.112	0.742	0.007	0.112	0.061
EvsN * PvsV	LPFC to RPFC	0.001	1	0.001	0.003	0.958	0.000	0.003	0.050
	LPFC to LPMC	1.826	1	1.826	5.197	0.038	0.257	5.197	0.569
	LPFC to RPMC	0.913	1	0.913	1.490	0.241	0.090	1.490	0.208
	LPFC to SMA	0.021	1	0.021	0.074	0.790	0.005	0.074	0.057
	RPFC to LPFC	0.032	1	0.032	0.064	0.803	0.004	0.064	0.056
	RPFC to LPMC	0.182	1	0.182	0.465	0.506	0.030	0.465	0.098
	RPFC to RPMC	0.263	1	0.263	0.746	0.401	0.047	0.746	0.128
	RPFC to SMA	0.028	1	0.028	0.104	0.751	0.007	0.104	0.061
	LPMC to LPFC	0.102	1	0.102	0.264	0.615	0.017	0.264	0.077
	LPMC to RPFC	2.132	1	2.132	8.523	0.011	0.362	8.523	0.779
	LPMC to RPMC	0.637	1	0.637	1.015	0.330	0.063	1.015	0.157
	LPMC to SMA	0.057	1	0.057	0.174	0.682	0.011	0.174	0.068
	RPMC to LPFC	0.169	1	0.169	0.818	0.380	0.052	0.818	0.135

	RPMC to RPFC	0.255	1	0.255	1.174	0.296	0.073	1.174	0.174
	RPMC to LPMC	0.444	1	0.444	0.814	0.381	0.051	0.814	0.135
	RPMC to SMA	1.009	1	1.009	3.007	0.103	0.167	3.007	0.368
	SMA to LPFC	1.723	1	1.723	6.824	0.020	0.313	6.824	0.685
	SMA to RPFC	0.294	1	0.294	1.181	0.294	0.073	1.181	0.175
	SMA to LPMC	0.503	1	0.503	1.364	0.261	0.083	1.364	0.194
	SMA to RPMC	0.034	1	0.034	0.204	0.658	0.013	0.204	0.071
Error	LPFC to RPFC	4.543	15	0.303					
	LPFC to LPMC	5.270	15	0.351					
	LPFC to RPMC	9.192	15	0.613					
	LPFC to SMA	4.216	15	0.281					
	RPFC to LPFC	7.578	15	0.505					
	RPFC to LPMC	5.861	15	0.391					
	RPFC to RPMC	5.289	15	0.353					
	RPFC to SMA	4.038	15	0.269					

	LPMC to LPFC	5.768	15	0.385					
	LPMC to RPFC	3.752	15	0.250					
	LPMC to RPMC	9.412	15	0.627					
	LPMC to SMA	4.912	15	0.327					
	RPMC to LPFC	3.100	15	0.207					
	RPMC to RPFC	3.258	15	0.217					
	RPMC to LPMC	8.173	15	0.545					
	RPMC to SMA	5.030	15	0.335					
	SMA to LPFC	3.788	15	0.253					
	SMA to RPFC	3.733	15	0.249					
	SMA to LPMC	5.531	15	0.369					
	SMA to RPMC	2.498	15	0.167					

a. Computed using alpha = 0.05

Supplementary Table 3: *Tests of Between-subjects effects*

Source	Dependent Variable	Type III Sum of Squares	df	Mean Square	F	Sig.	Partial Eta Squared	Noncent. Parameter	Observed Power[u]

Corrected Model	CoV-LPFC to RPFC	0.241[a]	3	0.080	1.135	0.358	0.139	3.404	0.261
	CoV-LPFC to LPMC	0.208[b]	3	0.069	1.759	0.186	0.201	5.277	0.391
	CoV-LPFC to RPMC	0.086[c]	3	0.029	0.392	0.760	0.053	1.176	0.114
	CoV-LPFC to SMA	0.347[d]	3	0.116	2.356	0.101	0.252	7.067	0.509
	CoV-RPFC to LPFC	0.367[e]	3	0.122	0.851	0.481	0.108	2.554	0.203
	CoV-RPFC to LPMC	0.068[f]	3	0.023	0.283	0.837	0.039	0.850	0.095
	CoV-RPFC to RPMC	0.027[g]	3	0.009	0.152	0.928	0.021	0.455	0.073
	CoV-RPFC to SMA	0.284[h]	3	0.095	1.336	0.289	0.160	4.008	0.303
	CoV-LPMC to LPFC	0.012[i]	3	0.004	0.055	0.983	0.008	0.164	0.058
	CoV-LPMC to RPFC	0.159[j]	3	0.053	0.581	0.634	0.077	1.743	0.150
	CoV-LPMC to RPMC	0.712[k]	3	0.237	3.009	0.053	0.301	9.028	0.623
	CoV-LPMC to SMA	0.457[l]	3	0.152	1.051	0.391	0.131	3.153	0.244
	CoV-RPMC to LPFC	0.085[m]	3	0.028	0.257	0.856	0.035	0.770	0.091
	CoV-RPMC to RPFC	0.485[n]	3	0.162	1.695	0.199	0.195	5.086	0.378
	CoV-RPMC to LPMC	0.042[o]	3	0.014	0.177	0.911	0.025	0.531	0.077

	CoV-RPMC to SMA	0.082[p]	3	0.027	0.195	0.899	0.027	0.585	0.080
	CoV-SMA to LPFC	0.065[q]	3	0.022	0.234	0.872	0.032	0.702	0.087
	CoV-SMA to RPFC	0.119[r]	3	0.040	0.501	0.685	0.067	1.504	0.134
	CoV-SMA to LPMC	0.107[s]	3	0.036	0.348	0.791	0.047	1.044	0.107
	CoV-SMA to RPMC	0.129[t]	3	0.043	0.665	0.583	0.087	1.995	0.166
Intercept	CoV-LPFC to RPFC	8.952	1	8.952	126.369	0.000	0.858	126.369	1.000
	CoV-LPFC to LPMC	9.630	1	9.630	243.842	0.000	0.921	243.842	1.000
	CoV-LPFC to RPMC	10.603	1	10.603	145.661	0.000	0.874	145.661	1.000
	CoV-LPFC to SMA	15.701	1	15.701	319.366	0.000	0.938	319.366	1.000
	CoV-RPFC to LPFC	9.942	1	9.942	69.188	0.000	0.767	69.188	1.000
	CoV-RPFC to LPMC	9.959	1	9.959	125.351	0.000	0.857	125.351	1.000
	CoV-RPFC to RPMC	9.745	1	9.745	166.516	0.000	0.888	166.516	1.000
	CoV-RPFC to SMA	15.810	1	15.810	223.142	0.000	0.914	223.142	1.000
	CoV-LPMC to LPFC	13.392	1	13.392	184.307	0.000	0.898	184.307	1.000
	CoV-LPMC to RPFC	13.154	1	13.154	143.779	0.000	0.873	143.779	1.000

	CoV-LPMC to RPMC	15.598	1	15.598	197.816	0.000	0.904	197.816	1.000
	CoV-LPMC to SMA	13.692	1	13.692	94.486	0.000	0.818	94.486	1.000
	CoV-RPMC to LPFC	14.890	1	14.890	134.185	0.000	0.865	134.185	1.000
	CoV-RPMC to RPFC	17.021	1	17.021	178.673	0.000	0.895	178.673	1.000
	CoV-RPMC to LPMC	11.651	1	11.651	146.699	0.000	0.875	146.699	1.000
	CoV-RPMC to SMA	12.089	1	12.089	86.393	0.000	0.804	86.393	1.000
	CoV-SMA to LPFC	12.033	1	12.033	130.525	0.000	0.861	130.525	1.000
	CoV-SMA to RPFC	8.971	1	8.971	113.133	0.000	0.843	113.133	1.000
	CoV-SMA to LPMC	14.279	1	14.279	138.914	0.000	0.869	138.914	1.000
	CoV-SMA to RPMC	8.793	1	8.793	135.759	0.000	0.866	135.759	1.000
EvsN	CoV-LPFC to RPFC	0.087	1	0.087	1.234	0.279	0.055	1.234	0.185
	CoV-LPFC to LPMC	0.062	1	0.062	1.576	0.223	0.070	1.576	0.224
	CoV-LPFC to RPMC	0.032	1	0.032	.441	0.514	0.021	0.441	0.097
	CoV-LPFC to SMA	0.221	1	0.221	4.488	0.046	0.176	4.488	0.524
	CoV-RPFC to LPFC	0.109	1	0.109	0.755	0.395	0.035	0.755	0.132

	CoV-RPFC to LPMC	0.040	1	0.040	0.501	0.487	0.023	0.501	0.104
	CoV-RPFC to RPMC	0.013	1	0.013	0.227	0.639	0.011	0.227	0.074
	CoV-RPFC to SMA	0.073	1	0.073	1.025	0.323	0.047	1.025	0.162
	CoV-LPMC to LPFC	0.007	1	0.007	0.103	0.752	0.005	0.103	0.061
	CoV-LPMC to RPFC	0.042	1	0.042	0.461	0.504	0.021	0.461	0.099
	CoV-LPMC to RPMC	0.000	1	0.000	0.006	0.940	0.000	0.006	0.051
	CoV-LPMC to SMA	0.371	1	0.371	2.559	0.125	0.109	2.559	0.333
	CoV-RPMC to LPFC	0.016	1	0.016	0.148	0.705	0.007	0.148	0.066
	CoV-RPMC to RPFC	0.041	1	0.041	0.433	0.518	0.020	0.433	0.096
	CoV-RPMC to LPMC	0.015	1	0.015	0.183	0.673	0.009	0.183	0.069
	CoV-RPMC to SMA	0.023	1	0.023	0.167	0.687	0.008	0.167	0.068
	CoV-SMA to LPFC	0.027	1	0.027	0.289	0.597	0.014	0.289	0.081
	CoV-SMA to RPFC	0.000	1	0.000	0.004	0.951	0.000	0.004	0.050
	CoV-SMA to LPMC	0.023	1	0.023	0.220	0.644	0.010	0.220	0.073
	CoV-SMA to RPMC	0.049	1	0.049	0.754	0.395	0.035	0.754	0.132

PvsV	CoV-LPFC to RPFC	0.001	1	0.001	0.015	0.904	0.001	0.015	0.052
	CoV-LPFC to LPMC	0.138	1	0.138	3.482	0.076	0.142	3.482	0.429
	CoV-LPFC to RPMC	0.023	1	0.023	0.322	0.577	0.015	0.322	0.084
	CoV-LPFC to SMA	0.106	1	0.106	2.161	0.156	0.093	2.161	0.289
	CoV-RPFC to LPFC	0.084	1	0.084	0.584	0.453	0.027	0.584	0.113
	CoV-RPFC to LPMC	0.000	1	0.000	0.003	0.958	0.000	0.003	0.050
	CoV-RPFC to RPMC	0.000	1	0.000	0.003	0.960	0.000	0.003	0.050
	CoV-RPFC to SMA	0.124	1	0.124	1.749	0.200	0.077	1.749	0.243
	CoV-LPMC to LPFC	0.003	1	0.003	0.035	0.853	0.002	0.035	0.054
	CoV-LPMC to RPFC	0.098	1	0.098	1.071	0.312	0.049	1.071	0.167
	CoV-LPMC to RPMC	0.047	1	0.047	0.597	0.448	0.028	0.597	0.114
	CoV-LPMC to SMA	0.003	1	0.003	0.021	0.885	0.001	0.021	0.052
	CoV-RPMC to LPFC	0.053	1	0.053	0.478	0.497	0.022	0.478	0.101
	CoV-RPMC to RPFC	0.429	1	0.429	4.498	0.046	0.176	4.498	0.525
	CoV-RPMC to LPMC	0.012	1	0.012	0.149	0.703	0.007	0.149	0.066

	CoV-RPMC to SMA	0.007	1	0.007	0.050	0.826	0.002	0.050	0.055
	CoV-SMA to LPFC	0.032	1	0.032	0.352	0.559	0.017	0.352	0.088
	CoV-SMA to RPFC	0.002	1	0.002	0.028	0.869	0.001	0.028	0.053
	CoV-SMA to LPMC	0.068	1	0.068	0.662	0.425	0.031	0.662	0.122
	CoV-SMA to RPMC	0.005	1	0.005	0.070	0.793	0.003	0.070	0.057
EvsN * PvsV	CoV-LPFC to RPFC	0.161	1	0.161	2.271	0.147	0.098	2.271	0.301
	CoV-LPFC to LPMC	0.004	1	0.004	0.100	0.755	0.005	0.100	0.061
	CoV-LPFC to RPMC	0.032	1	0.032	0.444	0.512	0.021	0.444	0.098
	CoV-LPFC to SMA	0.015	1	0.015	0.313	0.582	0.015	0.313	0.083
	CoV-RPFC to LPFC	0.182	1	0.182	01.268	0.273	0.057	1.268	0.189
	CoV-RPFC to LPMC	0.025	1	0.025	0.309	0.584	0.014	0.309	0.083
	CoV-RPFC to RPMC	0.014	1	0.014	0.242	0.628	0.011	0.242	0.076
	CoV-RPFC to SMA	0.067	1	0.067	0.945	0.342	0.043	0.945	0.153
	CoV-LPMC to LPFC	0.002	1	0.002	0.028	0.870	0.001	0.028	0.053
	CoV-LPMC to RPFC	0.019	1	0.019	0.212	0.650	0.010	0.212	0.072

	CoV-LPMC to RPMC	0.675	1	0.675	8.561	0.008	0.290	8.561	0.797
	CoV-LPMC to SMA	0.098	1	0.098	0.673	0.421	0.031	0.673	0.123
	CoV-RPMC to LPFC	0.017	1	0.017	0.154	0.699	0.007	0.154	0.066
	CoV-RPMC to RPFC	0.030	1	0.030	0.310	0.583	0.015	0.310	0.083
	CoV-RPMC to LPMC	0.013	1	0.013	0.160	0.694	0.008	0.160	0.067
	CoV-RPMC to SMA	0.051	1	0.051	0.364	0.552	0.017	0.364	0.089
	CoV-SMA to LPFC	0.008	1	0.008	0.082	0.777	0.004	0.082	0.059
	CoV-SMA to RPFC	0.118	1	0.118	1.488	0.236	0.066	1.488	0.214
	CoV-SMA to LPMC	0.018	1	0.018	0.177	0.678	0.008	0.177	0.069
	CoV-SMA to RPMC	0.081	1	0.081	1.245	0.277	0.056	1.245	0.187
Error	CoV-LPFC to RPFC	1.488	21	0.071					
	CoV-LPFC to LPMC	0.829	21	0.039					
	CoV-LPFC to RPMC	1.529	21	0.073					
	CoV-LPFC to SMA	1.032	21	0.049					
	CoV-RPFC to LPFC	3.018	21	0.144					

	CoV-RPFC to LPMC	1.668	21	0.079					
	CoV-RPFC to RPMC	1.229	21	0.059					
	CoV-RPFC to SMA	1.488	21	0.071					
	CoV-LPMC to LPFC	1.526	21	0.073					
	CoV-LPMC to RPFC	1.921	21	0.091					
	CoV-LPMC to RPMC	1.656	21	0.079					
	CoV-LPMC to SMA	3.043	21	0.145					
	CoV-RPMC to LPFC	2.330	21	0.111					
	CoV-RPMC to RPFC	2.001	21	0.095					
	CoV-RPMC to LPMC	1.668	21	0.079					
	CoV-RPMC to SMA	2.938	21	0.140					
	CoV-SMA to LPFC	1.936	21	0.092					
	CoV-SMA to RPFC	1.665	21	0.079					
	CoV-SMA to LPMC	2.159	21	0.103					
	CoV-SMA to RPMC	1.360	21	0.065					

Total	CoV-LPFC to RPFC	10.754	25						
	CoV-LPFC to LPMC	10.572	25						
	CoV-LPFC to RPMC	12.224	25						
	CoV-LPFC to SMA	16.938	25						
	CoV-RPFC to LPFC	13.273	25						
	CoV-RPFC to LPMC	11.645	25						
	CoV-RPFC to RPMC	11.049	25						
	CoV-RPFC to SMA	17.373	25						
	CoV-LPMC to LPFC	15.016	25						
	CoV-LPMC to RPFC	15.405	25						
	CoV-LPMC to RPMC	18.235	25						
	CoV-LPMC to SMA	17.181	25						
	CoV-RPMC to LPFC	17.301	25						
	CoV-RPMC to RPFC	19.675	25						
	CoV-RPMC to LPMC	13.319	25						

	CoV-RPMC to SMA	15.080	25						
	CoV-SMA to LPFC	14.117	25						
	CoV-SMA to RPFC	10.863	25						
	CoV-SMA to LPMC	16.534	25						
	CoV-SMA to RPMC	10.323	25						
Corrected Total	CoV-LPFC to RPFC	1.729	24						
	CoV-LPFC to LPMC	1.038	24						
	CoV-LPFC to RPMC	1.614	24						
	CoV-LPFC to SMA	1.380	24						
	CoV-RPFC to LPFC	3.385	24						
	CoV-RPFC to LPMC	1.736	24						
	CoV-RPFC to RPMC	1.256	24						
	CoV-RPFC to SMA	1.772	24						
	CoV-LPMC to LPFC	1.538	24						
	CoV-LPMC to RPFC	2.081	24						

	CoV-LPMC to RPMC	2.368	24						
	CoV-LPMC to SMA	3.500	24						
	CoV-RPMC to LPFC	2.416	24						
	CoV-RPMC to RPFC	2.485	24						
	CoV-RPMC to LPMC	1.710	24						
	CoV-RPMC to SMA	3.020	24						
	CoV-SMA to LPFC	2.001	24						
	CoV-SMA to RPFC	1.785	24						
	CoV-SMA to LPMC	2.266	24						
	CoV-SMA to RPMC	1.489	24						

Supplementary Table 4: *Tests of between-subjects effects*

Measure: MEASURE_1

Transformed Variable: Average

Source	Type III Sum of Squares	df	Mean Square	F	Sig.	Partial Eta Squared
Intercept	3065861.833	1	3065861.833	721.963	0.000	0.973
EvsN	54296.382	1	54296.382	12.786	0.002	0.390
PvsV	5509.986	1	5509.986	1.298	0.268	0.061
EvsN * PvsV	6320.988	1	6320.988	1.488	0.237	0.069

Error	84931.245	20	4246.562			

Supplementary Table 5: *Tests of between-subjects effects*

Dependent Variable: FLSCV

Source	Type III Sum of Squares	df	Mean Square	F	Sig.	Partial Eta Squared
Corrected Model	0.078[a]	3	0.026	2.268	0.110	0.245
Intercept	0.541	1	0.541	47.011	0.000	0.691
EvsN	0.050	1	0.050	4.370	0.049	0.172
PvsV	0.004	1	0.004	0.385	0.542	0.018
EvsN * PvsV	0.019	1	0.019	1.662	0.211	0.073
Error	0.242	21	0.012			
Total	0.838	25				
Corrected Total	0.320	24				

Supplementary Table 6: *ANOVA*

Model		Sum of Squares	df	Mean Square	F	Sig.
1	Regression	41423.157	21	1972.531	1.156	0.307[b]
	Residual	162038.691	95	1705.670		
	Total	203461.848	116			
2	Regression	41410.546	20	2070.527	1.227	0.250[c]
	Residual	162051.302	96	1688.034		
	Total	203461.848	116			
3	Regression	41346.583	19	2176.136	1.302	0.200[d]
	Residual	162115.264	97	1671.291		
	Total	203461.848	116			

4	Regression	41220.084	18	2290.005	1.383	0.157[e]
	Residual	162241.764	98	1655.528		
	Total	203461.848	116			
5	Regression	41137.719	17	2419.866	1.476	0.119[f]
	Residual	162324.128	99	1639.638		
	Total	203461.848	116			
6	Regression	41021.877	16	2563.867	1.578	0.089[g]
	Residual	162439.971	100	1624.400		
	Total	203461.848	116			
7	Regression	40806.342	15	2720.423	1.689	0.065[h]
	Residual	162655.506	101	1610.451		
	Total	203461.848	116			
8	Regression	40589.854	14	2899.275	1.816	0.046[i]
	Residual	162871.994	102	1596.784		
	Total	203461.848	116			
9	Regression	40244.893	13	3095.761	1.954	0.032[j]
	Residual	163216.955	103	1584.631		
	Total	203461.848	116			
10	Regression	39798.192	12	3316.516	2.107	0.022[k]
	Residual	163663.656	104	1573.689		
	Total	203461.848	116			
11	Regression	39293.188	11	3572.108	2.285	0.015[l]
	Residual	164168.659	105	1563.511		
	Total	203461.848	116			
12	Regression	38594.560	10	3859.456	2.481	0.010[m]

	Residual	164867.287	106	1555.352		
	Total	203461.848	116			
13	Regression	37961.178	9	4217.909	2.727	0.007[n]
	Residual	165500.669	107	1546.735		
	Total	203461.848	116			
14	Regression	37068.047	8	4633.506	3.007	0.004[o]
	Residual	166393.800	108	1540.683		
	Total	203461.848	116			
15	Regression	35841.987	7	5120.284	3.330	0.003[p]
	Residual	167619.861	109	1537.797		
	Total	203461.848	116			
16	Regression	34894.318	6	5815.720	3.795	0.002[q]
	Residual	168567.529	110	1532.432		
	Total	203461.848	116			
17	Regression	33916.412	5	6783.282	4.441	0.001[r]
	Residual	169545.435	111	1527.436		
	Total	203461.848	116			
18	Regression	32779.523	4	8194.881	5.377	0.001[s]
	Residual	170682.325	112	1523.949		
	Total	203461.848	116			
19	Regression	30682.228	3	10227.409	6.689	0.000[t]
	Residual	172779.619	113	1529.023		
	Total	203461.848	116			
20	Regression	27745.248	2	13872.624	9.000	0.000[u]
	Residual	175716.600	114	1541.374		

Total	203461.848	116			

a. Dependent Variable: FLSscore

b. Predictors: (Constant), Trials, C11, C1, C5, C9, C7, C12, C17, C2, C6, C14, C18, C20, C10, C13, C4, C8, C19, C16, C15, C3

c. Predictors: (Constant), Trials, C11, C1, C5, C9, C7, C12, C17, C2, C6, C14, C18, C20, C10, C13, C4, C8, C19, C16, C15

d. Predictors: (Constant), Trials, C11, C5, C9, C7, C12, C17, C2, C6, C14, C18, C20, C10, C13, C4, C8, C19, C16, C15

e. Predictors: (Constant), Trials, C11, C5, C9, C7, C12, C2, C6, C14, C18, C20, C10, C13, C4, C8, C19, C16, C15

f. Predictors: (Constant), Trials, C11, C5, C9, C7, C12, C2, C6, C14, C18, C20, C10, C13, C4, C8, C16, C15

g. Predictors: (Constant), Trials, C11, C9, C7, C12, C2, C6, C14, C18, C20, C10, C13, C4, C8, C16, C15

h. Predictors: (Constant), Trials, C11, C9, C7, C12, C2, C6, C14, C18, C20, C13, C4, C8, C16, C15

i. Predictors: (Constant), Trials, C11, C9, C7, C2, C6, C14, C18, C20, C13, C4, C8, C16, C15

j. Predictors: (Constant), Trials, C11, C9, C7, C2, C6, C14, C20, C13, C4, C8, C16, C15

k. Predictors: (Constant), Trials, C11, C9, C7, C2, C6, C14, C20, C13, C8, C16, C15

l. Predictors: (Constant), Trials, C11, C9, C7, C2, C6, C14, C20, C13, C8, C16

m. Predictors: (Constant), Trials, C9, C7, C2, C6, C14, C20, C13, C8, C16

n. Predictors: (Constant), Trials, C9, C7, C6, C14, C20, C13, C8, C16

o. Predictors: (Constant), Trials, C7, C6, C14, C20, C13, C8, C16

p. Predictors: (Constant), Trials, C6, C14, C20, C13, C8, C16

q. Predictors: (Constant), Trials, C6, C14, C13, C8, C16

r. Predictors: (Constant), Trials, C6, C14, C13, C8

s. Predictors: (Constant), Trials, C6, C14, C8

t. Predictors: (Constant), Trials, C14, C8

u. Predictors: (Constant), Trials, C8

Supplementary Table 7: *ANOVA*

Model		Sum of Squares	df	Mean Square	F	Sig.
1	Regression	0.219	20	0.011	0.432	0.907[b]
	Residual	0.101	4	0.025		
	Total	0.320	24			
2	Regression	0.219	19	0.012	0.568	0.831[c]
	Residual	0.101	5	0.020		
	Total	0.320	24			
3	Regression	0.219	18	0.012	0.720	0.728[d]
	Residual	0.101	6	0.017		
	Total	0.320	24			
4	Regression	0.219	17	0.013	0.888	0.608[e]
	Residual	0.101	7	0.014		
	Total	0.320	24			
5	Regression	0.216	16	0.013	1.034	0.506[f]
	Residual	0.104	8	0.013		
	Total	0.320	24			
6	Regression	0.213	15	0.014	1.201	0.402[g]
	Residual	0.107	9	0.012		
	Total	0.320	24			
7	Regression	0.212	14	0.015	1.397	0.302[h]
	Residual	0.108	10	0.011		

	Total	0.320	24			
8	Regression	0.209	13	0.016	1.586	0.225[i]
	Residual	0.111	11	0.010		
	Total	0.320	24			
9	Regression	0.203	12	0.017	1.731	0.177[j]
	Residual	0.117	12	0.010		
	Total	0.320	24			
10	Regression	0.197	11	0.018	1.895	0.136[k]
	Residual	0.123	13	0.009		
	Total	0.320	24			
11	Regression	0.189	10	0.019	2.011	0.113[l]
	Residual	0.131	14	0.009		
	Total	0.320	24			
12	Regression	0.183	9	0.020	2.221	0.083[m]
	Residual	0.137	15	0.009		
	Total	0.320	24			
13	Regression	0.172	8	0.022	2.328	0.071[n]
	Residual	0.148	16	0.009		
	Total	0.320	24			
14	Regression	0.159	7	0.023	2.412	0.065[o]
	Residual	0.161	17	0.009		
	Total	0.320	24			
15	Regression	0.153	6	0.025	2.740	0.045[p]
	Residual	0.167	18	0.009		
	Total	0.320	24			

16	Regression	0.138	5	0.028	2.878	0.042[q]
	Residual	0.182	19	0.010		
	Total	0.320	24			
17	Regression	0.121	4	0.030	3.053	0.041[r]
	Residual	0.199	20	0.010		
	Total	0.320	24			
18	Regression	0.099	3	0.033	3.140	0.047[s]
	Residual	0.221	21	0.011		
	Total	0.320	24			
19	Regression	0.084	2	0.042	3.912	0.035[t]
	Residual	0.236	22	0.011		
	Total	0.320	24			

a. Dependent Variable: FLSCV

b. Predictors: (Constant), C20CV, C8CV, C12CV, C18CV, C4CV, C7CV, C9CV, C17CV, C2CV, C3CV, C11CV, C5CV, C19CV, C15CV, C1CV, C6CV, C16CV, C14CV, C13CV, C10CV

c. Predictors: (Constant), C20CV, C8CV, C12CV, C18CV, C4CV, C7CV, C9CV, C17CV, C2CV, C3CV, C11CV, C5CV, C19CV, C15CV, C1CV, C6CV, C16CV, C13CV, C10CV

d. Predictors: (Constant), C20CV, C8CV, C12CV, C18CV, C4CV, C7CV, C9CV, C17CV, C2CV, C11CV, C5CV, C19CV, C15CV, C1CV, C6CV, C16CV, C13CV, C10CV

e. Predictors: (Constant), C20CV, C8CV, C12CV, C18CV, C4CV, C7CV, C9CV, C17CV, C2CV, C11CV, C5CV, C19CV, C15CV, C6CV, C16CV, C13CV, C10CV

f. Predictors: (Constant), C20CV, C8CV, C12CV, C18CV, C4CV, C7CV, C9CV, C17CV, C2CV, C11CV, C5CV, C19CV, C15CV, C16CV, C13CV, C10CV

g. Predictors: (Constant), C20CV, C8CV, C12CV, C18CV, C4CV, C7CV, C9CV, C17CV, C2CV, C11CV, C5CV, C19CV, C15CV, C16CV, C10CV

h. Predictors: (Constant), C20CV, C8CV, C12CV, C18CV, C4CV, C9CV, C17CV, C2CV, C11CV, C5CV, C19CV, C15CV, C16CV, C10CV

i. Predictors: (Constant), C20CV, C8CV, C12CV, C18CV, C9CV, C17CV, C2CV, C11CV, C5CV, C19CV, C15CV, C16CV, C10CV

j. Predictors: (Constant), C20CV, C8CV, C12CV, C18CV, C9CV, C17CV, C2CV, C5CV, C19CV, C15CV, C16CV, C10CV

k. Predictors: (Constant), C20CV, C8CV, C12CV, C18CV, C9CV, C17CV, C2CV, C5CV, C15CV, C16CV, C10CV

l. Predictors: (Constant), C20CV, C12CV, C18CV, C9CV, C17CV, C2CV, C5CV, C15CV, C16CV, C10CV

m. Predictors: (Constant), C12CV, C18CV, C9CV, C17CV, C2CV, C5CV, C15CV, C16CV, C10CV

n. Predictors: (Constant), C12CV, C18CV, C9CV, C17CV, C5CV, C15CV, C16CV, C10CV

o. Predictors: (Constant), C12CV, C9CV, C17CV, C5CV, C15CV, C16CV, C10CV

p. Predictors: (Constant), C9CV, C17CV, C5CV, C15CV, C16CV, C10CV

q. Predictors: (Constant), C9CV, C17CV, C5CV, C15CV, C16CV

r. Predictors: (Constant), C9CV, C17CV, C5CV, C15CV

s. Predictors: (Constant), C9CV, C5CV, C15CV

t. Predictors: (Constant), C9CV, C15CV

Supplementary Table 8: *Shapiro–Wilk's test of normality for the Granger causality measures*

ANOVA1 for each trial: Expert Physical (α = 0.01)						
S.N.	Connections	Trail-1	Trail-2	Trail-3	Trail-4	Trail-5
1	LPFC-->RPFC	0.2255	0.8764	0.9759	0.6713	0.9943
2	LPFC-->LPMC	0.2058	0.6884	0.2401	0.5285	0.9844
3	LPFC-->RPMC	0.2025	0.9087	0.1312	0.8479	0.9513
4	LPFC-->SMA	0.9203	0.5900	0.4026	0.8405	0.0981
5	RPFC-->LPFC	0.1536	0.5431	0.6759	0.1096	0.1867
6	RPFC-->LPMC	0.8566	0.4197	0.8651	0.3578	0.6280
7	RPFC-->RPMC	0.7969	0.3372	0.8951	0.5191	0.0724
8	RPFC-->SMA	0.4268	0.3986	0.5002	0.4201	0.0404
9	LPMC-->LPFC	0.2815	0.0961	0.3827	0.9771	0.2977
10	LPMC-->RPFC	0.1462	0.6913	0.4905	0.9688	0.7016
11	LPMC-->RPMC	0.9615	0.8018	0.0287	0.1975	0.3955
12	LPMC-->SMA	0.3986	0.6846	0.2905	0.8460	0.7455
13	RPMC-->LPFC	0.1273	0.3251	0.4836	0.8892	0.4926
14	RPMC-->RPFC	0.8153	0.3955	0.5046	0.7227	0.9002
15	RPMC-->LPMC	0.8455	0.9088	0.9134	0.1073	0.7666
16	RPMC-->SMA	0.7763	0.6253	0.4321	0.9080	0.2233
17	SMA-->LPFC	0.7919	0.8047	0.1840	0.9291	0.6686
18	SMA-->RPFC	0.6888	0.7166	0.6810	0.0775	0.3682
19	SMA-->LPMC	0.6049	0.4352	0.7633	0.3397	0.7187
20	SMA-->RPMC	0.7168	0.1884	0.9378	0.4940	0.8654

Table A: p value of ANOVA test for experts on the physical simulator

ANOVA1 for each trial: Novice Physical (α = 0.01)						
S.N.	Connections	Trail-1	Trail-2	Trail-3	Trail-4	Trail-5
1	LPFC-->RPFC	0.8766	0.8591	0.3533	0.5517	0.4045
2	LPFC-->LPMC	0.0966	0.2750	0.2944	0.5150	0.6971
3	LPFC-->RPMC	0.4802	0.2211	0.8651	0.4623	0.8536
4	LPFC-->SMA	0.2678	0.9002	0.6538	0.9253	0.8766
5	RPFC-->LPFC	0.6071	0.2064	0.5789	0.9168	0.3863
6	RPFC-->LPMC	0.1899	0.9968	0.9245	0.0444	0.4532
7	RPFC-->RPMC	0.5731	0.4675	0.6396	0.9565	0.3200
8	RPFC-->SMA	0.1225	0.8302	0.9398	0.4585	0.8325
9	LPMC-->LPFC	0.0729	0.3484	0.8732	0.4909	0.4629
10	LPMC-->RPFC	0.3325	0.7951	0.7366	0.2626	0.5383
11	LPMC-->RPMC	0.6072	0.5871	0.5272	0.7831	0.7570
12	LPMC-->SMA	0.3052	0.6145	0.5684	0.9869	0.2780
13	RPMC-->LPFC	0.1714	0.6506	0.3683	0.9521	0.8897
14	RPMC-->RPFC	0.9123	0.3243	0.7781	0.8930	0.9570
15	RPMC-->LPMC	0.6508	0.6398	0.5584	0.4575	0.9670
16	RPMC-->SMA	0.0745	0.8825	0.1830	0.8210	0.2711
17	SMA-->LPFC	0.7659	0.8060	0.4495	0.9165	0.7892
18	SMA-->RPFC	0.4411	0.9571	0.5983	0.8898	0.4406
19	SMA-->LPMC	0.9025	0.9061	0.5566	0.9360	0.5744
20	SMA-->RPMC	0.4244	0.8756	0.8124	0.7678	0.8552

Table B: p value of ANOVA test for novices on the physical simulator

ANOVA1 for each trial: Expert Virtual (α = 0.01)						
S.N.	Connections	Trail-1	Trail-2	Trail-3	Trail-4	Trail-5
1	LPFC-->RPFC	0.1260	0.0945	0.2817	0.2609	0.4421
2	LPFC-->LPMC	0.6969	0.0823	0.1685	0.5108	0.4538
3	LPFC-->RPMC	0.6605	0.7930	0.5610	0.3201	0.7313
4	LPFC-->SMA	0.8113	0.4583	0.9117	0.4026	0.5387
5	RPFC-->LPFC	0.9383	0.6645	0.8560	0.3653	0.7511
6	RPFC-->LPMC	0.3041	0.1998	0.3834	0.3298	0.2517
7	RPFC-->RPMC	0.8762	0.0167	0.8768	0.6044	0.7757
8	RPFC-->SMA	0.9637	0.4417	0.3397	0.9356	0.1819
9	LPMC-->LPFC	0.6072	0.1589	0.3763	0.1632	0.2279
10	LPMC-->RPFC	0.8325	0.8702	0.0972	0.4255	0.1432
11	LPMC-->RPMC	0.6942	0.7115	0.9953	0.9945	0.8149
12	LPMC-->SMA	0.0632	0.0511	0.5119	0.2812	0.0494
13	RPMC-->LPFC	0.2972	0.3366	0.1482	0.2279	0.8844
14	RPMC-->RPFC	0.0991	0.2192	0.4301	0.9832	0.2910
15	RPMC-->LPMC	0.6767	0.3766	0.1478	0.6114	0.9395
16	RPMC-->SMA	0.8250	0.0021	0.9828	0.8522	0.6076
17	SMA-->LPFC	0.4899	0.9851	0.8862	0.5541	0.0644
18	SMA-->RPFC	0.7559	0.0719	0.2268	0.9380	0.2449
19	SMA-->LPMC	0.4721	0.8337	0.6606	0.6143	0.6197
20	SMA-->RPMC	0.0516	0.8539	0.2781	0.9794	0.2754

Table C: p value of ANOVA test for experts on the virtual simulator

ANOVA1 for each trial: Novice Virtual (α = 0.01)						
S.N.	Connections	Trail-1	Trail-2	Trail-3	Trail-4	Trail-5
1	LPFC-->RPFC	0.805	0.768	0.931	0.450	0.824
2	LPFC-->LPMC	0.670	0.956	0.275	0.084	0.864
3	LPFC-->RPMC	0.991	0.857	0.054	0.497	0.308
4	LPFC-->SMA	0.365	0.547	0.158	0.488	0.330
5	RPFC-->LPFC	0.505	0.951	0.611	0.260	0.670
6	RPFC-->LPMC	0.768	0.463	0.259	0.876	0.540
7	RPFC-->RPMC	0.597	0.652	0.234	0.893	0.047
8	RPFC-->SMA	0.613	0.292	0.760	0.984	0.902
9	LPMC-->LPFC	0.401	0.042	0.602	0.430	0.352
10	LPMC-->RPFC	0.602	0.055	0.182	0.420	0.183
11	LPMC-->RPMC	0.667	0.868	0.524	0.868	0.906
12	LPMC-->SMA	0.606	0.670	0.258	0.947	0.595
13	RPMC-->LPFC	0.049	0.336	0.144	0.496	0.336
14	RPMC-->RPFC	0.387	0.319	0.936	0.180	0.350
15	RPMC-->LPMC	0.484	0.793	0.774	0.726	0.798
16	RPMC-->SMA	0.861	0.571	0.272	0.466	0.321
17	SMA-->LPFC	0.314	0.843	0.043	0.087	0.043
18	SMA-->RPFC	0.989	0.860	0.785	0.076	0.304
19	SMA-->LPMC	0.924	0.426	0.750	0.831	0.938
20	SMA-->RPMC	0.613	0.462	0.613	0.042	0.043

Table D: p value of ANOVA test for novices on the virtual simulator

FLS pattern cutting task video

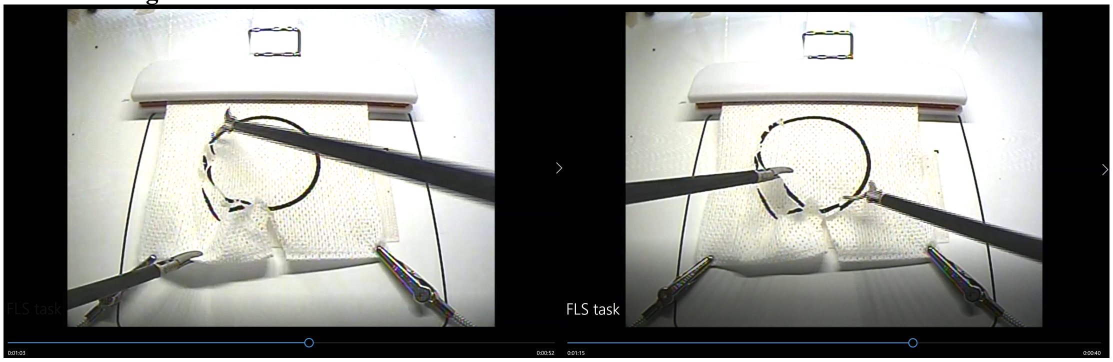

Video frames showing switching from left side cutting (left panel) to right side cutting (right panel) during an FLS pattern cutting task.

Granger Causality description

Consider two time series, $X_1(t)$ and $X_2(t)$, where we assume each of these time series can be modeled by the combination of one another as expressed below:

$$X_1(t) = \sum_{i=1}^{n} a_i X_1(t-i) + \sum_{i=1}^{n} b_i X_2(t-i) + \epsilon_1(t) \tag{1}$$

$$X_2(t) = \sum_{i=1}^{n} c_i X_1(t-i) + \sum_{i=1}^{n} d_i X_2(t-i) + \epsilon_2(t) \tag{2}$$

where n is the order of the process; a, b, c and d are the coefficients; and $\epsilon(t)$ is the additive prediction error. If we introduce a lag operator, L^k, such that the following equation is obtained:

$$L^k X(t) = X(t-k)$$

Then, we can rewrite Equations (1) and (2) as follows:

$$X_1(t) = \left(\sum_{i=1}^{n} a_i L^i\right) X_1(t) + \left(\sum_{i=1}^{n} b_i L^i\right) X_2(t) + \epsilon_1(t) \tag{3}$$

$$X_2(t) = \left(\sum_{i=1}^{n} c_i L^i\right) X_1(t) + \left(\sum_{i=1}^{n} d_i L^i\right) X_2(t) + \epsilon_2(t) \tag{4}$$

Equations (3) and (4) can be arranged in a matrix form as follows:

$$\begin{pmatrix} a(L) & b(L) \\ c(L) & d(L) \end{pmatrix} \begin{pmatrix} X_1(t) \\ X_2(t) \end{pmatrix} = \begin{pmatrix} \epsilon_1(t) \\ \epsilon_2(t) \end{pmatrix} \tag{5}$$

To work in the frequency domain, we transform Equation (5) using the fast Fourier transform (FFT) and obtain the following equation:

$$\begin{pmatrix} a(\omega) & b(\omega) \\ c(\omega) & d(\omega) \end{pmatrix} \begin{pmatrix} X_1(\omega) \\ X_2(\omega) \end{pmatrix} = \begin{pmatrix} \epsilon_1(\omega) \\ \epsilon_2(\omega) \end{pmatrix} \tag{6}$$

Where ω, is the frequency. We can rewrite Equation 6 as follows:

$$\begin{pmatrix} X_1(\omega) \\ X_2(\omega) \end{pmatrix} = \begin{pmatrix} H_{11}(\omega) & H_{12}(\omega) \\ H_{21}(\omega) & H_{22}(\omega) \end{pmatrix} \begin{pmatrix} \epsilon_1(\omega) \\ \epsilon_2(\omega) \end{pmatrix} \tag{7}$$

where $\boldsymbol{H}$ is the transfer matrix.

Then, GC can be found from the transfer matrix $\boldsymbol{H}$ and the spectral matrix $\boldsymbol{S}(\omega)$, which are related as follows [132]:

$$\boldsymbol{S}(\omega) = \boldsymbol{H}(\omega)\boldsymbol{\Sigma}(\omega)\boldsymbol{H}^*(\omega) \tag{8}$$

where * is the transposed conjugate operator. Here, the Wilson algorithm can factorize the spectral matrix, $\boldsymbol{S}(\omega)$, which is obtained from FFT as follows:

$$\begin{bmatrix} S_{11}(\omega) & S_{12}(\omega) \\ S_{21}(\omega) & S_{22}(\omega) \end{bmatrix} = \begin{bmatrix} H_{11}(\omega) & H_{12}(\omega) \\ H_{21}(\omega) & H_{22}(\omega) \end{bmatrix} \begin{bmatrix} \Sigma_{11}(\omega) & \Sigma_{12}(\omega) \\ \Sigma_{21}(\omega) & \Sigma_{22}(\omega) \end{bmatrix} \begin{bmatrix} H^*{}_{11}(\omega) & H^*{}_{12}(\omega) \\ H^*{}_{21}(\omega) & H^*{}_{22}(\omega) \end{bmatrix}$$

However, if $\sum_{12}(\omega) > 0$, then there is a third term resulting from the influence that correlated noise exerts on the spectra, which can be removed by the transformation introduced by Geweke [133]. This transformation is used here (indicated by a tilde); see [132,133] for details. Using Geweke's transformation and expanding Equation (8), the first term of $\boldsymbol{S}(\omega)$ can be written as follows:

$$S_{11}(\omega) = \widetilde{H}_{11}(\omega)\Sigma_{11}(\omega)\widetilde{H}^*_{11}(\omega) + H\widetilde{H}_{12}(\omega)\left(\Sigma_{22} - \frac{\Sigma^2_{12}}{\Sigma_{11}}\right)\widetilde{H}^*_{12}(\omega) \tag{9}$$

The first term of Equation (9) is the intrinsic term, and the second is the causal influence from the X_2 to X_1 term [133]. Therefore, the strict Granger causality from X_2 to X_1 ($I_{2\to1}$) can be defined by dividing by the intrinsic term as follows:

$$I_{2\to1} = \log\left(\frac{S_{11}(\omega)}{H_{11}(\omega)\Sigma_{11}H^*{}_{11}(\omega)}\right) \tag{10}$$

Following similar steps, the Granger causality from X_1 to X_2 ($I_{1\to2}$) is expressed as follows:

$$I_{1\to2} = \log\left(\frac{S_{22}(\omega)}{H_{22}(\omega)\Sigma_{22}H^*{}_{22}(\omega)}\right) \tag{11}$$

Finally, the GC values were integrated over the frequency range of 0.01 ($f1$) to 0.07 ($f2$) as follows:

$$GC_{i\to j} = \frac{1}{f_2 - f_1}\int_{f_1}^{f_2} I_{i\to j}(f)df$$

For our analysis, we selected 5 independent brain regions. To ensure that our variables are independent, we used Cholesky factorization before estimating the GC.

MATLAB code for Benjamini & Hochberg/Yekutieli false discovery rate control procedure for a set of statistical tests [David Groppe (2022). fdr_bh (https://www.mathworks.com/MATLABcentral/fileexchange/27418-fdr_bh), MATLAB Central File Exchange].

```
% fdr_bh() - Executes the Benjamini & Hochberg (1995) and the Benjamini &
%           Yekutieli (2001) procedure for controlling the false discovery
%           rate (FDR) of a family of hypothesis tests. FDR is the expected
%           proportion of rejected hypotheses that are mistakenly rejected
%           (i.e., the null hypothesis is actually true for those tests).
%           FDR is a somewhat less conservative/more powerful method for
%           correcting for multiple comparisons than other procedures, such as Bonferroni
%           correction, that provide strong control of the familywise
%           error rate (i.e., the probability that one or more null
%           hypotheses are mistakenly rejected).
%
%           This function also returns the false coverage-statement rate
%           (FCR)-adjusted selected confidence interval coverage (i.e.,
%           the coverage needed to construct multiple comparison corrected
%           confidence intervals that correspond to the FDR-adjusted p values).
%
%
% Usage:
%  >> [h, crit_p, adj_ci_cvrg, adj_p]=fdr_bh(pvals,q, method,report);
%
% Required Input:
%   pvals - A vector or matrix (two dimensions or more) containing the
%           p value of each individual test in a family of tests.
%
% Optional Inputs:
%   q       - The desired false discovery rate. {default: 0.05}
%   method  - ['pdep' or 'dep'] If 'pdep,' the original Benjamini & Hochberg
%             FDR procedure is used, which is guaranteed to be accurate if
%             the individual tests are independent or positively dependent
%             (e.g., Gaussian variables that are positively correlated or
%             independent). If 'dep,' the FDR procedure
%             described in Benjamini & Yekutieli (2001) that is guaranteed
%             to be accurate for any test dependency structure (e.g.,
%             Gaussian variables with any covariance matrix) is used. 'dep'
%             is always appropriate to use but is less powerful than 'pdep.'
%             {default: 'pdep'}
%   report  - ['yes' or 'no'] If 'yes', a brief summary of FDR results are
%             output to the MATLAB command line {default: 'no'}
%
%
% Outputs:
%   h       - A binary vector or matrix of the same size as the input "pvals."
%             If the ith element of h is 1, then the test that produced the
%             ith p value in pvals is significant (i.e., the null hypothesis
%             of the test is rejected).
%   crit_p  - All uncorrected p values less than or equal to crit_p are
%             significant (i.e., their null hypotheses are rejected). If
%             no p values are significant, crit_p=0.
%   adj_ci_cvrg - The FCR-adjusted BH- or BY-selected
%             confidence interval coverage. For any p values that
%             are significant after FDR adjustment, this gives the
```

```
%           proportion of coverage (e.g., 0.99) that should be used when generating
%           confidence intervals for those parameters. Specifically,
%           this allows one to correct your confidence intervals for
%           multiple comparisons. Confidence intervals cannot be obtained
%           for nonsignificant p values. The adjusted confidence intervals
%           guarantee that the expected FCR is less than or equal to q
%           if using the appropriate FDR control algorithm for the
%           dependency structure of your data (Benjamini & Yekutieli, 2005).
%           FCR (i.e., false coverage-statement rate) is the proportion
%           of confidence intervals you construct
%           that miss the true value of the parameter. adj_ci=NaN if no
%           p values are significant after adjustment.
%   adj_p   - All adjusted p values less than or equal to q are significant
%           (i.e., their null hypotheses are rejected). Note, adjusted
%           p values can be greater than 1.
%
%
% References:
%   Benjamini, Y. & Hochberg, Y. (1995) Controlling the false discovery
%     rate: A practical and powerful approach to multiple testing. Journal
%     of the Royal Statistical Society, Series B (Methodological). 57(1),
%     289-300.
%
%   Benjamini, Y. & Yekutieli, D. (2001) The control of the false discovery
%     rate in multiple testing under dependency. The Annals of Statistics.
%     29(4), 1165-1188.
%
%   Benjamini, Y., & Yekutieli, D. (2005). False discovery rate?adjusted
%     multiple confidence intervals for selected parameters. Journal of the
%     American Statistical Association, 100(469), 71?81. doi:10.1198/016214504000001907
%
%
% Example:
%  nullVars=randn(12,15);
%  [~, p_null]=ttest(nullVars); %15 tests where the null hypothesis
%  %is true
%  effectVars=randn(12,5)+1;
%  [~, p_effect]=ttest(effectVars); %5 tests where the null
%  %hypothesis is false
%  [h, crit_p, adj_ci_cvrg, adj_p]=fdr_bh([p_null p_effect].05,'pdep','yes');
%  data=[nullVars effectVars];
%  fcr_adj_cis=NaN*zeros(2,20); %initialize confidence interval bounds to NaN
%  if ~isnan(adj_ci_cvrg),
%     sigIds=find(h);
%     fcr_adj_cis(:,sigIds)=tCIs(data(:,sigIds), adj_ci_cvrg); % tCIs.m is available on the
%     %Mathworks File Exchange
%  end
%
%
% For a review of false discovery rate control and other contemporary
% techniques for correcting for multiple comparisons see:
%
%   Groppe, D.M., Urbach, T.P., & Kutas, M. (2011) Mass univariate analysis
% of event-related brain potentials/fields I: A critical tutorial review.
% Psychophysiology, 48(12) pp. 1711-1725, DOI: 10.1111/j.1469-8986.2011.01273.x
% http://www.cogsci.ucsd.edu/~dgroppe/PUBLICATIONS/mass_uni_preprint1.pdf
```

```
%
%
% For a review of FCR-adjusted confidence intervals (CIs) and other techniques
% for adjusting CIs for multiple comparisons see:
%
%   Groppe, D.M. (in press) Combating the scientific decline effect with
% confidence (intervals). Psychophysiology.
% http://biorxiv.org/content/biorxiv/early/2015/12/10/034074.full.pdf
%
%
% Author:
% David M. Groppe
% Kutaslab
% Dept. of Cognitive Science
% University of California, San Diego
% March 24, 2010

%%%%%%%%%%%%%%%% REVISION LOG %%%%%%%%%%%%%%%%%
%
% 5/7/2010-Added FDR adjusted p values
% 5/14/2013- D.H.J. Poot, Erasmus MC, improved run-time complexity
% 10/2015- Now returns FCR adjusted confidence intervals

function [h, crit_p, adj_ci_cvrg, adj_p]=fdr_bh(pvals,q, method, report)

if nargin<1,
    error('You need to provide a vector or matrix of p values. ');
else
    if ~isempty(find(pvals<0,1)),
        error('Some p values are less than 0. ');
    elseif ~isempty(find(pvals>1,1)),
        error('Some p values are greater than 1. ');
    end
end

if nargin<2,
q=.05;
end

if nargin<3,
    method='pdep';
end

if nargin<4,
    report='no';
end

s=size(pvals);
if (length(s)>2) || s(1)>1,
    [p_sorted, sort_ids]=sort(reshape(pvals,1,prod(s)));
else
    %p values are already a row vector
    [p_sorted, sort_ids]=sort(pvals);
end
[dummy, unsort_ids]=sort(sort_ids); %indices to return p_sorted to pvals order
m=length(p_sorted); %number of tests
```

```
if strcmpi(method,'pdep'),
    %BH procedure for independence or positive dependence
    thresh=(1:m)*q/m;
    wtd_p=m*p_sorted./(1:m);

elseif strcmpi(method,'dep')
    %BH procedure for any dependency structure
    denom=m*sum(1./(1:m));
    thresh=(1:m)*q/denom;
    wtd_p=denom*p_sorted./[1:m];
    %Note, it can produce adjusted p values greater than 1!
    %compute adjusted p values
else
    error('Argument "method" needs to be "pdep" or "dep". ');
end

if nargout>3,
    %compute adjusted p values; This can be slightly computationally intensive
    adj_p=zeros(1,m)*NaN;
    [wtd_p_sorted, wtd_p_sindex] = sort(wtd_p);
    nextfill = 1;
    for k = 1: m
        if wtd_p_sindex(k)>=nextfill
            adj_p(nextfill:wtd_p_sindex(k)) = wtd_p_sorted(k);
            nextfill = wtd_p_sindex(k)+1;
            if nextfill>m
                break;
            end;
        end;
    end;
    adj_p=reshape(adj_p(unsort_ids),s);
end

rej=p_sorted<=thresh;
max_id=find(rej,1,'last'); %find greatest significant pvalue
if isempty(max_id),
    crit_p=0;
    h=pvals*0;
    adj_ci_cvrg=NaN;
else
    crit_p=p_sorted(max_id);
    h=pvals<=crit_p;
    adj_ci_cvrg=1-thresh(max_id);
end

if strcmpi(report,'yes'),
    n_sig=sum(p_sorted<=crit_p);
    if n_sig==1,
        fprintf('Out of %d tests, %d is significant using a false discovery rate of %f.\n',m,n_sig,q);
    else
        fprintf('Out of %d tests, %d are significant using a false discovery rate of %f.\n',m,n_sig,q);
    end
    if strcmpi(method,'pdep'),
        fprintf('FDR/FCR procedure used is guaranteed valid for independent or positively dependent tests.\n');
    else
```

```
        fprintf('FDR/FCR procedure used is guaranteed valid for independent or dependent tests.\n');
    end
end
```
